# Perfluoroalkyl Substances in the Western Tropical Atlantic Ocean

**DOI:** 10.1021/acs.est.1c01794

**Published:** 2021-10-07

**Authors:** Daniele de A. Miranda, Juliana Leonel, Jonathan P. Benskin, Jana Johansson, Vanessa Hatje

**Affiliations:** † Centro Interdisciplinar de Energia & Ambiente (CIEnAm) and Inst. de Química, Universidade Federal da Bahia, 41170-115 Salvador, BA, Brazil; ‡ Department of Environmental Science, Stockholm University, Stockholm SE-106 91, Sweden; § Coordenação de Oceanografia, Universidade Federal de Santa Catarina, 88040-900 Florianópolis, SC, Brazil

**Keywords:** POPs, PFAAs, Upwelling, GEOTRACES, Tropical Atlantic Ocean

## Abstract

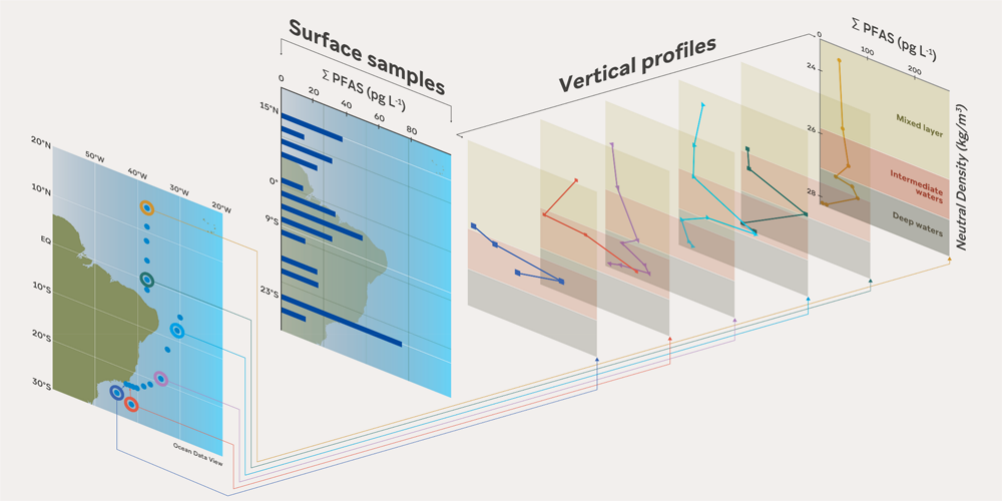

The dispersion of perfluoroalkyl substances (PFAS) in surface and deep-water profiles (down to 5845 m deep) was evaluated through the Western Tropical Atlantic Ocean (TAO) between 15°N and 23°S. The sum concentrations for eight quantifiable PFAS (∑_8_PFAS) in surface waters ranged from 11 to 69 pg/L, which is lower than previously reported in the same area as well as in higher latitudes. Perfluoroalkyl carboxylic acids (PFCAs) were the predominant PFASs present in the Western TAO. The 16 surface samples showed variable PFAS distributions, with the predominance of perfluorooctanoic acid (PFOA) along the transect (67%; 11 ± 8 pg/L) and detection of perfluoroalkyl sulfonic acids (PFSAs) only in the Southern TAO. Perfluoroheptanoic acid (PFHpA) was often detected in the vertical profiles. PFAS distribution patterns (i.e., profiles and concentrations) varied with depth throughout the TAO latitudinal sectors (North, Equator, South Atlantic, and in the Brazilian coastal zone). Vertical profiles in coastal samples displayed decreasing PFAS concentrations with increasing depth, whereas offshore samples displayed higher PFAS detection frequencies in the intermediate water masses. Together with the surface currents and coastal upwelling, the origin of the water masses was an important factor in explaining PFAS concentrations and profiles in the TAO.

## Abstract 2

PFAS observed in the Tropical Atlantic Ocean reflect ongoing production over several decades and subsequent distribution via oceanic long-range transport.

## Introduction

Perfluoroalkyl substances (PFAS) are fully fluorinated organic compounds that have been used since the 1950s^1^ but which only emerged as a new class of global pollutants in the early 2000s.^2^ Their unique physical–chemical properties (including stability, amphipathicity, and high surface activity) have made them attractive for use in a wide range of products.^3^ However, these properties also contribute to their environmental persistence, bioaccumulation, and global environmental occurrence.^4−8^ Moreover, the detection of PFAS in humans and wildlife is of considerable concern considering the links between PFAS exposure and adverse health effects.^9,10^

PFAS may be released into the environment from either direct use or via transformation of PFAS precursors.^11,12^ Long-range transport occurs both via the atmosphere (gas phase and/or on particles)^13−16^ and by oceanic currents.^17−21^ Furthermore, PFAS can cycle between the oceans and the atmosphere via sea spray aerosol.^22^ Some PFAS have been suggested as useful tracers of ocean circulation, similar to other contaminants such as tritium, chlorofluorocarbons (CFCs), and lead.^23−27^ Previous studies reported high PFAS concentrations in the North Atlantic and Arctic oceans and biota^28,29^ associated with production and usage of PFAS in the USA and Europe. PFAS have also been detected in the Equatorial and Southern Atlantic waters.^17−20,29−31^ Long-range oceanic transport is an important source of PFAS to the Southern Atlantic Ocean,^17,32^ considering the relatively small regional inputs previously observed there.^33−35^ However, a potential local source of perfluorooctanesulfonic acid (PFOS) to the South Atlantic Ocean is the use of the PFOS precursor formicide sulfluramid (*N*-ethyl perfluorooctane sulfonamide; EtFOSA) in South America, mainly in Brazil.^18,19^ The production and use of PFAS has changed over the past decades. Regulatory measures restricted the use of PFOS in 2009 and prohibited the use and production of perfluorooctanoic acid (PFOA) in 2018,^36^ the latter being the most widespread PFAS in seawater.^17,28,29,37^ The progressive elimination of PFAS that occurred decades ago is already reflected in the profiles and concentrations of these compounds found in surface water from different oceans.^21^

Although some studies have been carried out in the Tropical Atlantic Ocean (TAO), most were limited to surface waters and/or the first few hundred meters of the water column.^18−20,29−31,38^ Large discrepancies in PFAS concentrations between studies have been observed for surface seawater in the western TAO.^18,19^ For example, Benskin et al.^19^ found ∑PFAS concentrations in the order of parts-per-quadrillion (ppq), while González-Gaya et al.^18^ measured the same compounds (i.e., PFOS and other sulfonic and carboxylic compounds) at parts-per-trillion (ppt) levels. The reason for differences between studies remains unclear; however, possible explanations include the time elapsed between sampling campaigns (four years), environmental conditions, or a punctual discharge of PFAS after the first study.

The present work studied the occurrence of 14 PFAS at various depths in coastal and oceanic waters of the Western Tropical Atlantic Ocean (15°N to 23°S) to identify sources, distribution patterns, transport routes, and fate of PFAS. To the best of our knowledge, only one prior study investigated the occurrence of PFAS in deep water (i.e., below 4000 m),^17^ but this was limited to the North Atlantic and only involved a few substances (i.e., PFOA, perfluorobutanesulfonic acids (PFBS), and PFOS). Thus, the current work builds on the existing body of PFAS data in the global oceans to better understand (1) the importance of surface currents in the spread of PFAS for latitudinal long-range transport in the ocean and (2) the processes affecting the vertical distribution of PFAS in the water column. In the present study, we tested how the distance to historical source regions of PFAS and the oceanographic mechanisms (e.g., upwelling, water masses movement, surface currents transportation) affect their concentrations in waters of TAO.

## Materials and Methods

### Sampling Campaign

**Figure 1:**
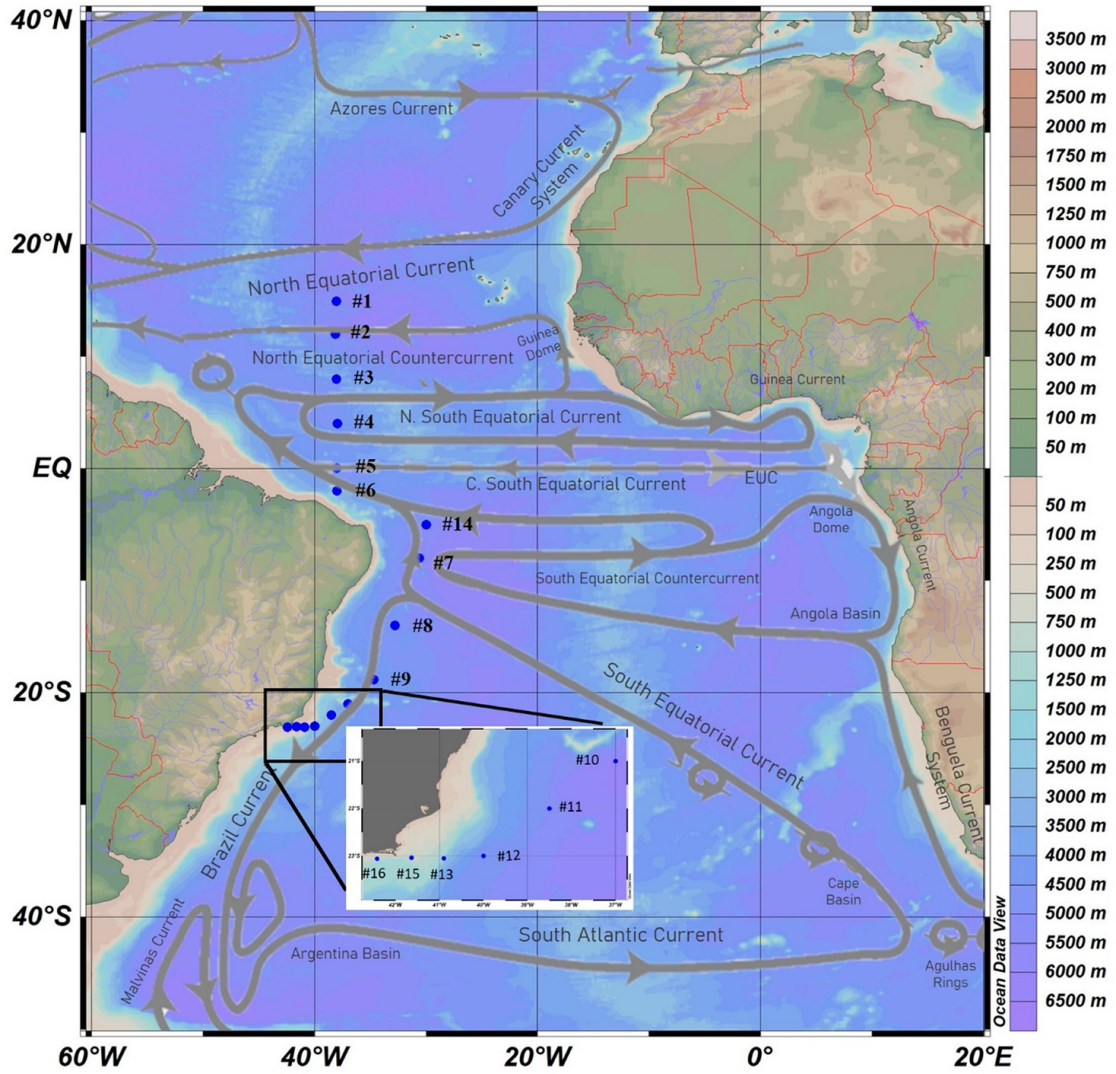
Location of sampling sites (#1–16) along the Western Tropical Atlantic Ocean. Deep water profiles were collected at #1, #5, #7, #9, #15, and #16. Gray arrows represent the main surface currents in the region (adapted from ref (43). Copyright 2017 Elsevier). The scale on the right represents depth.

Sixteen surface water (5–14 m deep) sites and six full depth (up to 5845 m deep) water profiles (Figure 1, Table S1) were sampled between November 2017 and January 2018 in the Tropical Atlantic Ocean (15°N to 23°S) on board of the R/V Vital de Oliveira, during the PIRATA XVII/GEOTRACES GApr10 cruise (http://pirata.ccst.inpe.br/, geotraces.org). Sampling locations were grouped in four zones: Northern TAO (samples #1–4), Equator (#5), Southern TAO (#6, #14, and #7 to #12), and coastal samples (#13, #15, and #16). Seawater samples were collected using NISKIN bottles accoupled to a CTD rosette sampler and were stored in 0.5 L polypropylene (PP) bottles. All sample bottles had been precleaned by rinsing 3 times with 1% ammonium hydroxide in methanol in a clean laboratory and then washing 3 times with seawater immediately prior to sampling. All samples were kept at 4 °C until analysis.

### Oceanographic Setting

#### Surface Circulation

##### Equatorial and Tropical Western Atlantic

Surface samples from #1.1 to #6.1 and #14.1 (where “1” behind the decimal denotes surface samples; Figure 1) are mostly influenced by Equatorial westward currents (Figure 1), being limited by the Equatorial flanks of the northern and southern subtropical gyres. While the North Equatorial Current (NEC) is fed by the Canary Current System and totally belongs to the Northern Hemisphere, the South Equatorial Current (SEC) is formed by three branches (South SEC, Central SEC and North SEC), with the first two branches crossing the South Atlantic at different latitudes under the influence of the Angola and the Benguela Current System. At the surface, the NEC and SEC are separated by the eastward flows of the North Equatorial Countercurrent (NECC) and the South Equatorial Countercurrent (SECC).

##### South Tropical Western Atlantic

The SEC reaches the Brazilian margin between 10° and 15°S (i.e., surface samples #14.1 and from #7.1 to #13.1, Figure 1), forming the northward North Brazil Current (NBC) and the southward Brazil Current (BC). BC is the Western Boundary Current that closes the South Atlantic subtropical gyre. This subtropical gyre is also composed of the eastward South Atlantic Current (SAC), followed by the Benguela Current and Agulhas waters flux intrusion and finally the SEC.

#### Water Masses

The thermohaline structure of the sampled transect is characterized vertically by different water masses (Figure S1, Table S2) that were seen along the depths in stations #1, #5, #7, and #9. Tropical Surface Waters (TW) are present in the mixed layer (top ∼100 m), which are characterized by warm and high salinity waters. Intermediate waters are represented by the North Atlantic Central Water (NACW) and South Atlantic Central Water (SACW), both presented between ∼200 and ∼600 m.^39−41^ NACW is formed at the south flank of the Gulf Stream,^41^ while SACW is formed by the confluence of Brazil and Malvinas currents at 35°S and by different modal waters, including the Southern Tropical Indian Modal Water (STIMW) and flows within the SAC until it merges into SEC at 10°S.^42,43^ Below the SACW, the Antarctic Intermediate Water (AAIW) extends from ∼600 m to more than 1200 m depth. In the Atlantic, it is formed near the Drake Passage, but the region also receives AAIW from the Indian Ocean through the Agulhas Current leakage; it is transported by the South Atlantic subtropical gyre.^39^ The North Atlantic Deep Water (NADW) is the following water mass occupying the depth of ∼1200–3500 m.^44^ This water mass is formed by the confluence of deep water in the Nordic and Labrador Seas and is then transported equatorward. Lastly, the Abyssal Antarctic Bottom Water (AABW) is originated in the Southern Ocean south of the Antarctic Circumpolar Current by brine rejection in the Weddell Sea.^43^ A summary of those water masses’ physicochemical characteristics can be seen in Table S2.

##### Cabo Frio, Rio de Janeiro Upwelling

At 23°S (stations #15 and #16), due to northeast strong winds, there is a modification in the coastal current direction from north–south to east–west that promotes the seasonal upwelling of the SACW to the photic zone of the continental shelf, being intensified during the summer.^45,46^

### Standards and Reagents

A total of 14 PFAS were investigated in this work, including 1 perfluoroalkyl sulfonamide (FASA), 9 perfluoroalkyl carboxylic acids (PFCAs), and 4 perfluoroalkyl sulfonic acids (PFSAs) (Table S3). Authentic standards of perfluooctane sulfonamide (FOSA), perfluorohexanoic acid (PFHxA), perfluoroheptanoic acid (PFHpA), PFOA, perfluorononanoic acid (PFNA), perfluorodecanoic acid (PFDA), perfluoroundecanoic acid (PFUnDA), perfluorododecanoic acid (PFDoDA), perfluorotridecanoic acid (PFTriDA), perfluorotetradecanoic acid (PFTeDA)), PFBS, perfluorohexanesulfonic acid (PFHxS), PFOS, and perfluorodecanesulfonic acid (PFDS) as well as the internal standards ^13^C_2_–PFHxA, ^13^C_4_–PFHpA, ^13^C_4_–PFOA, ^13^C_5_–PFNA, ^13^C_2_–PFDA, ^13^C_2_–PFUnDA, ^13^C_2_–PFDoDA, ^18^O_2_–PFHxS, ^13^C_4_–PFOS, and ^13^C_8_–FOSA were purchased from Wellington Laboratories (Guelph, ON, Canada).

### Sample Treatment

Samples (*n* = 51) were extracted using a method previously described in Gilljam et al.^47^ Briefly, isotopically labeled internal standards (100 pg) were added to each sample at least 24 h prior to extraction to allow the internal standard to equilibrate with the seawater sample. Seawater samples were analyzed without filtering as filtering is known to contribute to losses due to filter sorption.^48^ Waters oasis weak-anion exchange (WAX) solid-phase extraction (SPE) cartridges (6 cm^3^, 150 mg, 30 μm) were used to extract samples in a clean laboratory. Samples were not extracted onboard due to uncertainties surrounding background levels on the ship and because field sampling materials have previously been shown to contribute to PFAS contamination in samples.^49^ Several prior studies also opted for on-shore extraction of PFAS from water samples.^19,38,50−52^ SPE cartridges were conditioned with 15 mL of 0.3% of NH_4_OH in methanol (MeOH) followed by 4.5 mL of 0.1 M formic acid in ultrapure Milli-Q water and then loaded with unfiltered seawater samples (0.5 L). After loading, the cartridges were washed with 5 mL of 20% MeOH in 0.1 M formic acid and 2 mL of 0.3% NH_4_OH in Milli-Q water. Samples were eluted with 3 mL of 0.3% NH_4_OH in MeOH. The walls of the PP bottles were rinsed with MeOH, and this additional solvent was extracted with the sample by SPE in order to recover any possible compounds adsorbed on the walls of the bottles. Extracts were evaporated under a gentle flow of nitrogen to a final volume of 100 μL and transferred to a high-density polyethylene (HDPE) microvial and then stored in fridge until analysis. No buffer or recovery standard was used to reduce the risk of adding PFAS which may occur as impurities.

### Instrumental Analysis

Instrumental analysis was carried out by ultraperformance liquid chromatography–tandem mass spectrometry using a Waters Acquity UPLC coupled to a Waters Xevo TQ-S triple quadrupole mass spectrometer operated in negative electrospray ionization, multiple reaction monitoring mode. Twenty microliter aliquots of each sample were chromatographed on a BEH C18 analytical column (2.1 × 50 mm, 1.7 μm particle size, Waters) operated at a flow rate of 0.4 mL min^–1^ using a mobile phase composition of 90% water/10% acetonitrile containing 2 mM ammonium acetate (solvent A) and 99% acetonitrile and 1% water containing 2 mM ammonium acetate (solvent B). The mobile phase gradient profile is shown in Table S4. A total of two precursor/product ion transitions were monitored per analyte (Table S5), one for quantification and the other for qualification. Quantitative determination of target PFAS was carried out either by isotope dilution or an internal standard approach using a linear calibration curve with 1/*x* weighting. Branched isomers were determined semiquantitatively using the calibration curve for the linear isomer. The data through the manuscript is presented as average ± SD.

### Quality Assurance/Quality control

A total of seven laboratory-, two bottle-, and three field blanks were included in this study to account for any potential contamination that could be introduced during sample collection and handling. Field blanks consisted of PP water bottles containing 1 mL of C18 SPE-extracted ultrapure water in Brazil (Universidade Federal da Bahia), transported to the field, and uncapped during sample collection. Bottle blanks were transported empty and kept sealed during the entire sampling collection and filled immediately prior to sample extraction with 500 mL of polished ultrapure water (i.e., ultrapure water that was passed through a conditioned WAX SPE cartridge) in Stockholm University, Sweden. Finally, a laboratory blank (1 mL of polished ultrapure water) was extracted together with each batch of 12 samples. All blanks were extracted in the same manner as real samples. Further details on optimization of the PFAS extraction method can be found in the Supporting Information.

Accuracy and precision for individual PFAS were assessed using replicate spike/recovery experiments (Table S6) at both a low- (50 pg individual PFAS in 500 mL Milli-Q water; *n* = 8) and high (500 pg of individual PFAS into 500 mL; *n* = 8) fortification level. Percent recoveries for the low-level fortification ranged from 70 and 92% for most substances, except PFTeDA and PFTriDA, which showed low recovery (27 ± 8%, 40 ± 12%, respectively), and PFBS, which showed higher recovery (124 ± 64%) (Table S6). Recoveries at the high fortification level were mostly between 60 and 110%, except for PFTeDA (34 ± 11%), PFBS (136 ± 124%), and FOSA (132 ± 84%). Method detection and quantification limits (MDLs and MQLs, respectively; Table S6) were calculated in samples as the average concentration of each compound producing a signal-to-noise ratio ≥3 and ≥10, respectively. MDLs ranged from 0.50 (L-PFOA) to 6.88 pg L^–1^ (PFTriDA), where MQLs were between 1.67 (PFDA/PFTeDA) to 22.9 pg L^–1^ (PFTriDA). Concentrations below MDL were not included in any PFAS sum, whereas concentrations between MDL and MQL were reported as measured, although these concentrations should be considered more uncertain. Individual PFAS concentrations in laboratory blanks were always <1 pg L^–1^. PFAS contamination in field blanks was also very low (<5 pg L^–1^), but slightly higher than lab blanks, possibly due to contamination introduced when preparing the field blanks (Table S7). Overall, blank contamination was very low and consistent; therefore, blank corrections was applied as an average of all three types of blanks (i.e., field, bottle, and lab blanks; *n* = 12) for PFHxA, PFDA, PFDoDA, and PFOS (see Table S7). PFHpA, PFOA (linear and branched), and PFHxS could not be reported for profile #1 (15°N) due to contamination of the reagents used for this batch of samples (Table S7). These reagents were removed when processing subsequent batches and did not affect any other samples.

## Results and Discussion

### PFAS in Surface Samples

**Figure 2:**
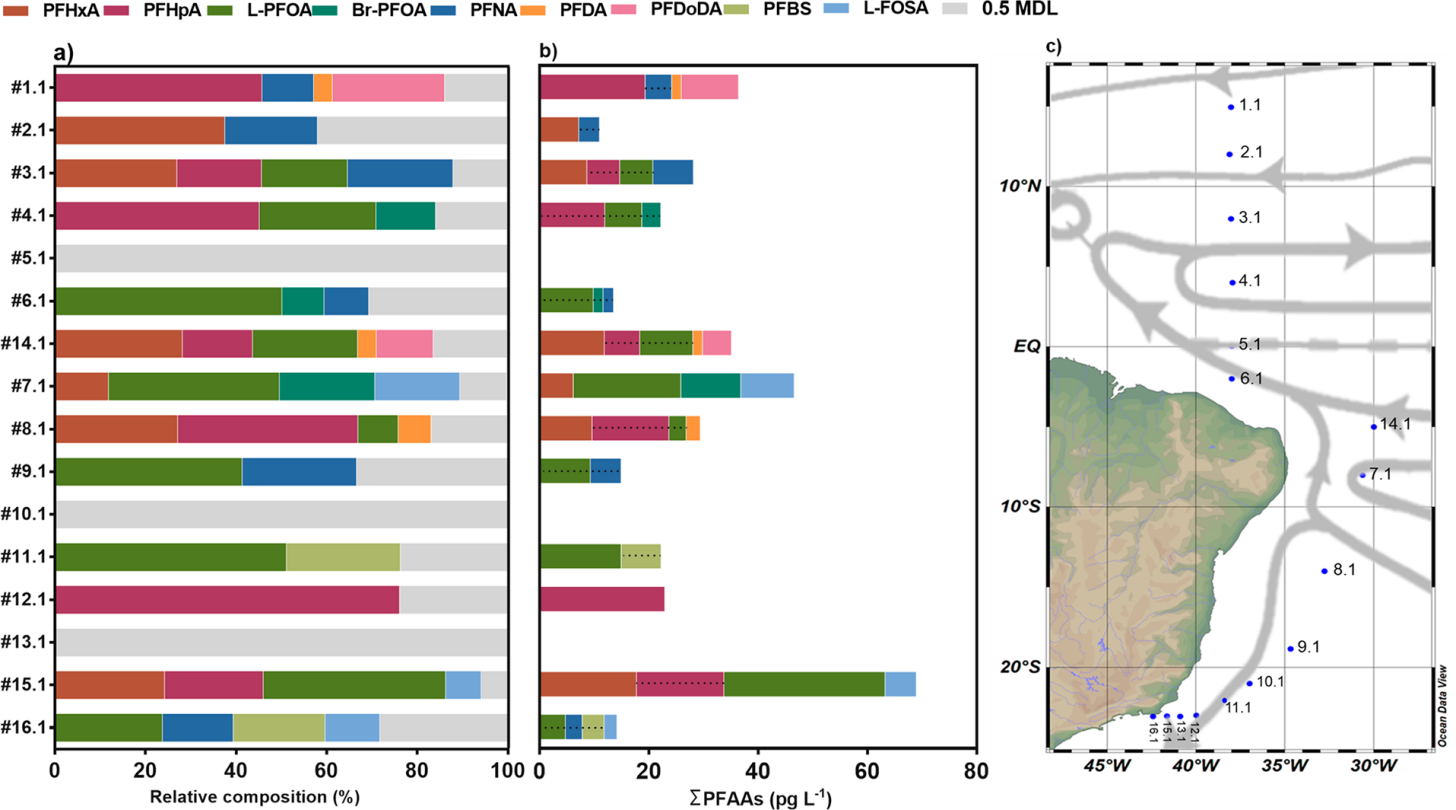
Relative composition (a) and the sum (∑) of PFAS (b) in surface waters of the Western Tropical Atlantic Ocean (c). Samples #5.1, #10.1, and #13.1 presented concentrations below detection limits for all compounds. Concentrations below the method detection limit (MDL) were not computed in the PFAS sum. PFHxA and PFOA (L- and Br-) were not reported for #1.1 due to analytical issues. Gray bars in (a) represent sum of half values of the MDL. Values above MDL and below method quantification limit are showed with a dashed line in (b). Gray arrows in (c) show the surface current directions.

Eight of the 14 PFAS investigated here (i.e., PFHxA, PFHpA, PFOA, PFNA, PFDA, PFDoDA, PFHxS, and FOSA) were detected in most of the surface water samples above the MDL (Table S8), with ΣPFAS concentrations ranging from 11 to 69 pg L^–1^ (Figure 2, Table S8). Concentrations in samples #5.1 (0°), #10.1 (21°S), and #13.1 (23°S) were below the MDL for all targets. No correlation was observed between latitude and ∑PFAS (rs = 0.055; *p* = 0.858). However, the PFAS profile varied along the latitudinal transect, reflecting different surface currents passing through the region (Figure 1). For example, L-PFHxA, which was frequently detected over the entire transect (40% detection frequency; 10 ± 4 pg L^–1^), is mainly associated with surface currents from around Africa (i.e., #2.1 and #3.1: Guinea Dome; #7.1 and #14.1: Angola Basin) as well as the South Equatorial Current (#8.1). This compound was reported in samples collected in 2005 at similar concentrations over the same latitude (i.e., 12°N to 22°S; 11 ± 10 pg L^–1^, *n* = 12 sampling points)^19^ and was also dominant in samples from the eastern TAO sampled in 2010 (13 ± 2 pg L^–1^).^20^

PFOA was the most frequently detected PFAS over the entire transect (67%). The observed levels (11 ± 8 pg L^–1^) were comparable to previous observations from the same latitude (i.e., 12°N to 25°S) collected in 2005 (21 ± 11 pg L^–1^, *n* = 12 sampling points)^19^ but lower than observations from 2008 and 2009^30,38^ in the eastern TAO (11° to 8°N, *n* = 3, 78 ± 9 pg L^–1^; and 15°N to 23°S, *n* = 7, 54 ± 10 pg L^–1^, respectively). Previous studies have reported PFOA concentrations below MDL (<4 and <12 pg L^–1^) in the southern Equator in 2008^30^ and 2010^20^ (Table S2), suggesting different sources for this region and the eastern TAO.

∑_6_PFAS (i.e., PFHxA, PFHpA, L- and Br-PFOA, PFNA, PFBS, and L-FOSA) concentrations observed throughout the TAO (range 11–69 pg L^–1^) are lower than those in roughly corresponding latitudes reported by Benskin et al.^19^ (∑_6_PFAS: 22 to 95 pg L^–1^) sampled in 2007 and Ahrens et al.^30^ (∑_6_PFAS: 27 to 126 pg L^–1^) in 2008 for the same compounds and orders of magnitude lower than concentrations in open oceans from high latitudes of the Northern Hemisphere.^20,29,53,54^ The lower PFAS concentrations detected in the TAO here and by previous studies compared to other locations were expected considering (1) the distance to the main PFAS sources regions (e.g., USA and Europe); (2) the progressive phase-out and voluntary changes in PFAS uses over the last two decades; (3) and the predominant direction of currents that move surface water from source regions northward to the Arctic, by the prolongation of the Gulf Stream current, a North Atlantic Current (NAC).^32,55^ Consequently, the southern surface branch of the NAC, driven toward the equator, carries lower amounts of PFAS than the northern branch.^30^ The southern branch of the NAC feeds the Canary Current system and, in turn, the North Equatorial system. The Canary Current spreads PFAS from Europe to Equatorial waters,^20,30^ and the source of compounds to this current system may include contaminants from the English Channel,^56^ North sea,^57^ and Bay of Biscay.^20^ These sources were previously reported as important contaminant contributors to the Canary Current system, showing similar PFAS profiles with a high contribution of PFCAs.

Sample #1.1 (15°N) showed the highest ∑PFAS concentrations among the surface samples in the Northern TAO (15°N; 36 pg L^–1^). The other samples from the northern TAO (#2.1–6.1) are less influenced by the North Equatorial current and more affected by the east–west Equatorial Currents, which seem to decrease ∑PFAS concentration (11 to 28 pg L^–1^) in the region. PFAS sources for the east–west Equatorial Currents are unclear but may include atmospheric deposition, in particular considering the elevated precipitation rate in this area^58^ and the lack of large inputs of PFAS reported for the western Africa coast.^30^ The atmospheric occurrence of PFCAs and PFSA-precursors (i.e., FASA, fluorotelomer alcohols [FTOHs], and others) was previously reported for the eastern Atlantic, showing a gradual concentration decrease southward (from ∼53°N to ∼33°S).^13,59^

PFSAs and perfluoroalkyl sulfonamides (PFBS: 6 ± 2 pg L^–1^; and FOSA: 6 ± 3 pg L^–1^) were only present in the southern TAO, in coastal waters (#15.1 and #16.1; 23°S) and in two samples offshore (#7.1 and #11.1; 8° and 22°S). In comparison, Benskin et al.^19^ observed higher detection frequency of these substances for the western TAO (Figure S2; FOSA 1–3 pg L^–1^), although FOSA concentrations were lower. While PFOS was not observed in surface samples here, previous studies conducted in 2007^19^ and 2011^18^ over a nearby area suggested the ongoing use of the Sulfluramid in South America, which may have contributed to the increase of ∑PFSAs in the Atlantic. To date, occurrence of PFAS in marine and estuarine waters of South America remains associated with multiple sources including Sulfluramid use in Brazilian forestry^33,47^ due to the environmental transformation (either biotic or abiotic) of this formicide to FOSA and PFOS.^60^

The transect in the continental shelf of the Rio de Janeiro (#13.1, #15.1, and #16.1; 23°S) showed different ∑PFAS concentrations and profiles toward the coast (Figure 2). While sample #15.1 (Figure 2) showed the highest concentration of PFAS (69 pg L^–1^) and the highest concentration of L-PFOA (30 pg L^–1^) in the entire transect, sample #16.1 showed the second lowest ∑PFAS concentration (14 pg L^–1^). The concentrations and PFAS profiles observed here were lower than previous studies carried out near urban areas in the North Atlantic^61^ and Pacific^62^ and different from other TAO zones in this study. Although the presence of PFAS in the TAO has been associated with riverine inputs,^33,63^ the lower PFAS concentration in #16.1 compared to #15.1 suggests an additional source of contaminants other than direct continental input to the latter sample. Nevertheless, PFOA detected here was previously observed in the main bay and river around the sampled area.^34^ Although atmospheric deposition can be an important contaminant source,^13,59,64^ the unique PFAS profile and levels in the region reinforce the influence of additional sources. According to the NOAA meteorological back air trajectories data (Figures S3 and S4), the study sites (i.e., #13.1, #15.1, and #16.1) are under the influence of the same air current, carrying continental air masses to the sampled area. If atmospheric deposition was the most important source for the region, a more homogeneous PFAS distribution would be expected in coastal samples. As such, continental inputs and the different hydrographic processes acting in the water column (i.e., upwelling) should be considered to explain the observed distribution patterns. More details on how the hydrographic processes can be acting to spread these compounds through the water column are given in the next section.

### PFAS in Deep Water Samples

**Figure 3:**
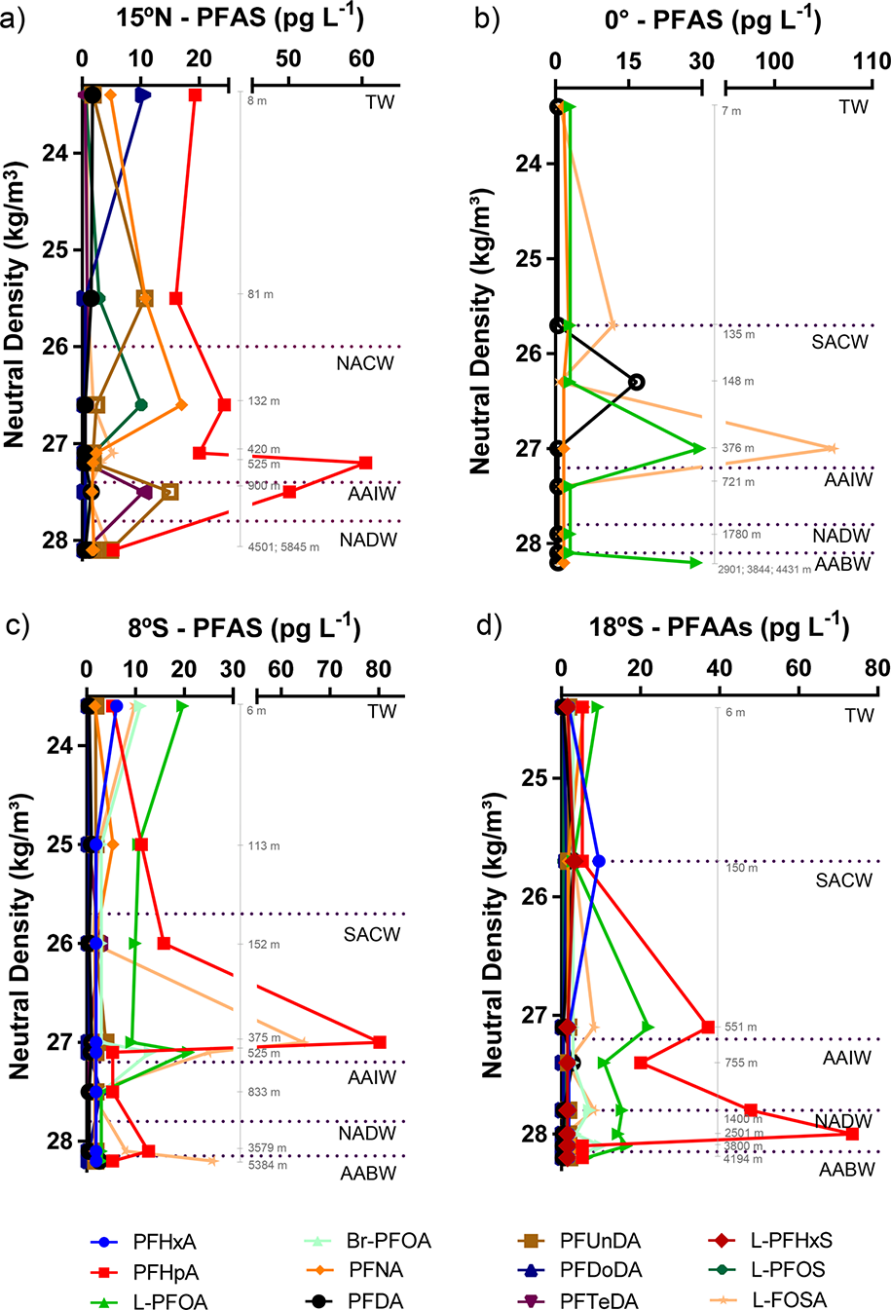
Vertical profiles of PFAS (pg L^–1^) in ocean water columns above MDL from Tropical Atlantic Ocean ((a)15°N [#1]; (b) 0° [#5]; (c) 8°S [#7]; and (d) 18°S [#9]) together with neutral density (kg/m^**3**^). PFHxA and PFOA (L- and Br-) were not reported for 15°N due to analytical issues. Dotted lines represent the water masses (TW: Tropical Water; NACW: North Atlantic Central Water; SACW: South Atlantic Central Water; AAIW: Antarctic Intermediate Water; mAAIW: modified AAIW; NADW: North Atlantic Deep Water; AABW: Antarctic Bottom Water). Values in gray represent the sampling depth.

**Figure 4:**
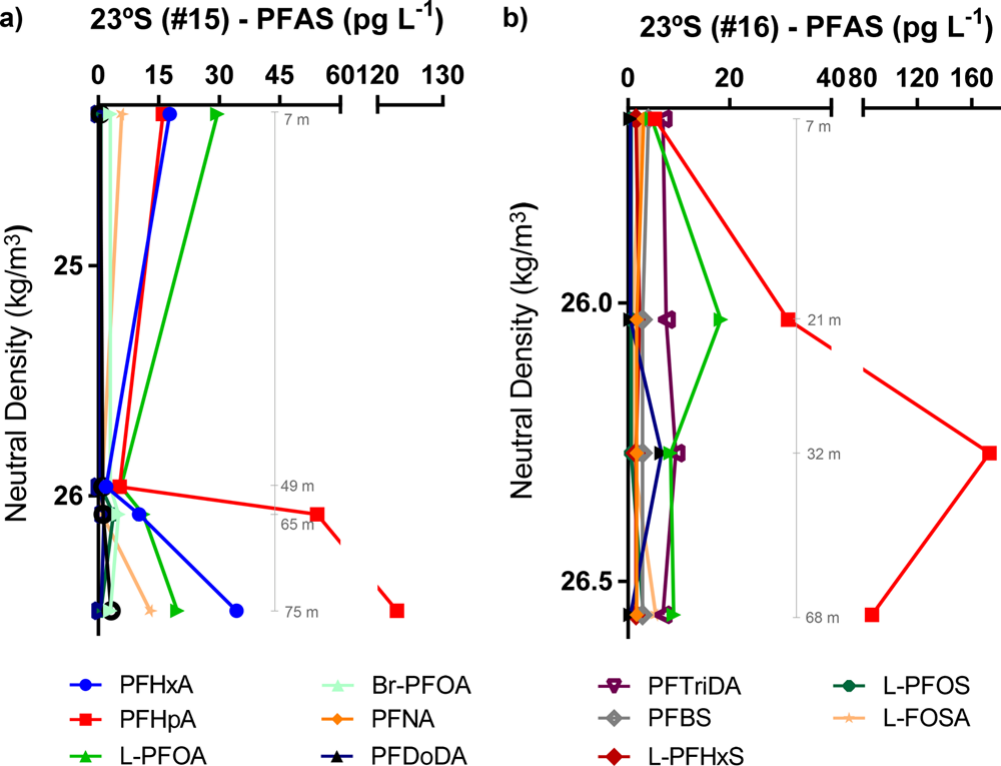
Vertical profiles of PFAS (pg L^–1^) in ocean water columns above MDL from Rio de Janeiro upwelling at 23°S ((a) #15 and (b) #16) together with neutral density (kg/m^3^). Values in gray represent the sampling depth.

Nine of 14 analyzed PFAS were detected in deep profiles (Figures 3 and 4, Table S8). The vertical PFAS patterns were different across TAO zones, except for the consistent predominance of long-chain PFCAs compared to PFSAs. Most compounds were detected in the mixed layer (TW) and intermediate central waters (both NACW and SACW, between ∼135 and ∼525 m deep) and, occasionally, in the bottom water (AABW) (Figure 3; individual PFAS profiles by depth: Figures S5 and S6). The vertical profile at station #9 (18°S) showed different distribution of compounds with the highest ∑PFAS in the NADW (81 and 92 pg L^–1^ at 1399 and 2500 m deep, respectively). The higher concentrations in intermediate waters are associated with the area of formation of these water masses and their trajectory in both hemispheres in the ocean surface;^39,65^ being exposed to PFAS inputs by both atmospheric deposition and from continental sources. Besides the horizontal water mass movement spreading compounds far away from the respective formation areas, the vertical process of biological sedimentation, that is important for transference of elements from surface to deep waters,^71^ should also be considered in the sinking of PFAS compounds in TAO from mixed layer to intermediate waters.^26,31,^

The NACW (15°N) showed a different profile from the SACW, with PFHpA, PFNA, and PFOS representing the dominant PFAS, at concentrations of 35 ± 18, 7 ± 7, and 6 ± 4 pg L^–1^ (average of samples in the same water mass ± SD), respectively (Figure 3, Table S8). The frequent occurrence of PFHpA has been previously observed in the Atlantic, Mediterranean, and Arctic surface waters,^19,54,66^ and its detection was suggested as an impurity in consumer products and degradation of fluorotelomer alcohols (FTOHs),^64^ whereas PFNA and PFOS were also associated with direct releases into the environment.^11^ The intensive historical use of PFAS-based products in the Northern Hemisphere explains the occurrence of PFHpA and PFNA in the NACW, whereas the rare detection and low concentrations of PFOS may reflect its progressive phase-out, which started in the early 2000s.^2^

In the Southern TAO, at 8°S (#7) and 18°S (#9), the SACW presented a larger number of compounds and higher concentrations than in the same water mass at the Equator (#5) (Figure 3, Table S8). Whereas PFOA was detected in the SACW and in similar concentrations among profiles (30, 13 ± 5, and 22 pg L^–1^; #5, #7, and #9, respectively), FOSA showed an increasing trend from 18°S northwards to the Equator (59 ± 47, 30 ± 26, and 6 ± 2 pg L^–1^, in samples #5, #7, and #9, respectively). The reason for these findings is unclear but is likely associated with different inputs to the individual sampling stations. PFHpA was observed in both stations in the southern hemisphere at SACW (#7 = 48 ± 32; and #9 = 37 pg L^–1^) and in the NACW (#1:35 ± 18 pg L^–1^). High PFAS concentrations in one of the seawater current sources for SACW (i.e., Malvinas current) had been previously associated with the inputs of Sulfluramid and other industrial and consumer products carried by the Plata River to the ocean.^19^ PFOA and PFOS were measured in surface waters by Benskin et al.^19^ in higher concentrations than observed here. While PFHpA and FOSA were rarely detected in that work; they were detected here in both deep profiles in the southern TAO. The SACW also receives input from the Indian Ocean via the Agulhas leakage, which is an important feature to the transference of heat and salts to the Atlantic Ocean,^67^ and should be considered as a potential source of PFAS.

PFAS were detected in AAIW in three of the four profiles along the TAO transect. The AAIW, formed in the Subantarctic Front between 50 and 60°S, crosses the Atlantic northward, transporting less saline water from the Southern to Northern Hemisphere.^68^ During its transport northward, AAIW follows the SAC, and similar to the SACW, AAIW also receives inputs from the Agulhas leakage.^42^ There are no previous studies analyzing vertical profiles in the Indian Ocean, but González-Gaya et al.^18^ observed similar PFAS in surface samples (i.e., PFHpA, PFOA, PFNA, PFOS, PFDA) to what was observed here, even though FOSA was not observed in this previous.^18^ The detection of FOSA in the present study may suggest additional source of PFAS compounds for both SACW and AAIW not elucidated here. In addition, recent study showed PFAS contribution from Africa rivers to the Indian ocean in ng L^–1^.^69^

Below the AAIW, the deep layer is occupied by the NADW. This water mass is formed by the sinking of surface waters in the Labrador Sea, which is a hotspot for PFAS.^17,19,32^ The mechanism of water formation in this region is well-known as an important pathway to remove PFAS from the ocean surface.^64^ This water mass is part of the overturning circulation and moves southward until it gets to the Antarctic at 60°S,^65^ being remarkable in the transport of cold water from the North to the South Hemisphere and for the spread of PFAS compounds in the same direction.^32^ The only study reporting PFAS in deep Atlantic waters, carried out in 2002, showed slightly higher concentrations of PFOA and PFOS than measured here (∼50 pg L^–1^ and ∼10 pg L^–1^, respectively), but in the same order of magnitude for stations in the Labrador Sea and Middle Atlantic Ocean (25°N).^17^ It was calculated that the CFC present in the NADW formation area (i.e., Labrador Sea, 47°N) reaches 20°S after ∼40 years.^66^ Considering the release of PFAS such as PFOA and PFOS from 1980 to 2000,^72^ the NADW formation area could be the source of PFHpA, (L- and Br-) PFOA, FOSA, PFOS and other PFAS found at latitude 18°S (#9). Combined, due to the release of PFAS in the last decades and the water mass movement southward, PFAS are expected to be still detected in NADW in the Southern Hemisphere for the coming years. The high concentrations associated with peak PFAS production appears to be moving southward (Figure 3). It is interesting to note the presence of Br-PFOA in this water mass, suggesting the input of this compound into the water masses by direct emission rather than precursor degradation.

The bottom water mass in the Atlantic is occupied by the AABW that showed only L-PFOA, above the MDL, in Equator (#5). L-PFOA was already observed in deep waters (∼5000 m) but at higher latitudes (25°N) and in lower concentrations.^17^ This water mass is formed by the abrupt sink of high saline and cold water in the Antarctic. Although PFAS have been measured in Antarctic snow in high concentrations,^20,51^ the detection of this compound at the interface between the NADW and AABW (i.e., 4500 m) is likely a combined signature of the former water masses. A possible explanation for this is mainly because there was not enough time for the contaminated waters in the Antarctic region to reach the sampling point at the Equator, due to the slower movement of this water mass when compared to NADW, and the contamination input in the formation area of the AABW (i.e., Weddell Sea) when compared to NADW (i.e., Labrador Sea).

### Cabo Frio, Rio de Janeiro Upwelling

The two vertical profiles sampled at the Brazilian continental shelf (stations #15 and #16, 23°S) contained PFAS profiles which were opposite to those observed in offshore areas, with high concentrations in the bottom samples (in particular in sample #15, 75 m but also #16, 68 m) and low concentrations in the surface (Figure 4). This result was unexpected due to the proximity to a highly industrialized and urbanized area, which was previously associated with high PFAS concentrations in surface waters.^62^ Despite their close proximity, the two profiles presented dissimilar concentrations, with ∑PFAS concentrations in #15 varying from 69 pg L^–1^ (#15.1; surface; 7 m) to 194 pg L^–1^ (#15.4; bottom; 75 m), while in #16, concentrations varied from 14 pg L^–1^ (#16.1; surface; 7) to 104 pg L^–1^ (#16.4; bottom; 68). This divergence could be due to physical processes occurring in the area. However, no significative difference was observed for ∑PFAS between the two profiles (t = −0.088; p = 0.051). This region is known for the occurrence of upwelling, which intensifies during the austral spring and summer (September to March) when the SACW is conducted from a depth of ∼300 m to the surface.^45^ Moreover, salinity, temperature, and oxygen profiles (Figures S7 and S8, Table S2) recorded the occurrence of recent upwelling events in #16 where SACW was outcropped, but the same was not observed in #15. SACW upwelling is induced when the NE trade winds push TW offshore explaining the lower concentrations in the station near the coast (#16) and higher concentrations in #15.

Previous data from river waters surrounding the upwelling stations showed L-PFOA, PFHpA, and PFOS among the most often detected compounds, with individual concentrations ranging from 150 to 3250, 111–1970, and 170–920 pg L^–1^, respectively.^34^ Together, the direct input by coastal water systems and the resuspension of contaminated particulate/sediments forced by the upwelling may be impacting PFAS distribution patterns observed in the deepest sampling points. Once in the seawater, these compounds will partition in different proportions to the particulate material according to their chemical structure, with long-chain compounds in greater affinity with the solid phase. A previous study showed the sediment signature of carbon–nitrogen ratio and their isotopes (i.e., C/N ratio and δ^13^C and δ^15^N) at the continental shelf of Rio de Janeiro associated with organic matter inflow from Paraiba do Sul River and at a lower degree from Guanabara.^70^ Thus, those factors must be investigated separately (i.e., PFAS sedimentation and upwelling) to better understand the influence of each forcing in more detail in the future.

## Overview and Future Perspectives

Surface and vertical profiles showed differences in the composition of PFAS, although PFCAs were the dominant group in both surface and deep waters. The absence of PFOS in surface waters and decreasing concentrations with increasing distance from emission sources in the Northern Hemisphere highlight the positive impacts of industry phase-outs and regulation of this chemical from 2000 to 2010. Also, the ongoing use of sulfluramid by South America was not observed to be an important source of PFOS to the TAO, since this compound was not found in mixed layers and intermediate water from southern TAO. However, the presence of FOSA (a Sulfluramid degradation product and PFOS precursor) in SACW in the southern TAO could raise concerns regarding historical uses of the formicide in the region and should be closely monitored. The composition of PFAS in the current investigation varied with depth, highlighting the contribution of different water masses in the stratification of these contaminants; however, these results must be interpreted cautiously due to the low concentrations and detection frequency of most PFAS in the TAO.

The widespread occurrence of PFAS in different areas of the TAO, as well as the diversity of PFAS found in the present study, reflects the persistence of these compounds and their diffuse sources, involving different production locations and uses. Water masses formed in hotspot regions are therefore expected to dominate the oceanic long-range transport of PFAS. Future studies should evaluate the various sources of PFAS to the TAO individually, with the aim of identification of the most important processes that lead to release and distribution of PFAS in oceanic environments. Such data would ultimately help to predict future contamination levels in this region. Finally, additional target PFAS should be included in future monitoring campaigns in order to assess the spread of these emerging replacement compounds.

## Supporting Information Available

Experimental details, Tables S1–-S9, and Figures S1–S9 (PDF)
