# Supplementary material for: Perfluoroalkyl Substances in the Western Tropical Atlantic Ocean
Source: Environ Sci Technol. 2021 Oct 7;55(20):13749–58. doi: 10.1021/acs.est.1c01794 (PMC8529868; doi:10.1021/acs.est.1c01794)
Supplement: Supplementary file 1 — es1c01794_si_001.pdf [file es1c01794_si_001.pdf]

Supplementary Information

Perfluoroalkyl Substances (PFAS) in the western Tropical Atlantic Ocean

Daniele de A. Miranda[a,b]*, Juliana Leonel[c], Jonathan P. Benskin[b] , Jana Johansson[b] , Vanessa Hatje[a]

[a]Centro Interdisciplinar de Energia & Ambiente (CIEnAm) and Inst. de Química, Universidade Federal da Bahia, 41170-115, Salvador, BA, Brazil;

[b]Department of Environmental Science, Stockholm University, Stockholm, Sweden.

[c]Coordenação de Oceanografia, Universidade Federal de Santa Catarina, Florianópolis, SC, Brazil.

*Corresponding author

Daniele Miranda (danielealmeida@ufba.br / Daniele.Miranda@aces.su.se)

Universidade Federal da Bahia

CIEnAm, Campus Ondina

Salvador, Bahia, Brazil. 41170-115

Table of Contents

Standard and reagent brands……………………………………………..……………..…………4

PFAS-extraction method optimization…………………………...……………………………..…..4

References………………………………………………………………………………..…..25

Tables

Table S1. Sample location, depth, temperature, salinity, oxygen and date of sampling. Pot. Temp.: Potential Temperature; Sal.: Salinity; Oxyg.: Oxygen; TW: Tropical Water; SACW: South Atlantic Central Water; AAIW: Antarctic Intermediate Water; mAAIW: modified AAIW; NADW: North Atlantic Deep Water; AABW: Antarctic Bottom Water. 5

Table S2. Water masses characteristics. Where = TW: Tropical Water; NACW: North Atlantic Central Water; SACW: South Atlantic Central Water; AAIW: Antarctic Intermediate Water; mAAIW: Mediterranean AAIW; NADW: North Atlantic Deep Water; AABW: Abyssal Antarctic Bottom Water. 7

Table S3. Perfluoroalkyl substances (PFAS) analyzed in the present study. 8

Table S4. Mobile phase gradient profile used in LC-MS/MS. 9

Table S5. List of retention times, and monitored qualitative and quantitative ions for each compound analyzed in the present study. 10

Table S6. Values for method detection limit (MDL), method quantification limit (MQL), and spike recovery used in the analytical analyses (n = 8, each). It is based on 500 mL extractions. Branched isomers MDL and MQL are the same as their linear isomers. 11

Table S7. Values for field (n = 3, 1 mL), lab (n = 7, 1 mL) bottle (n = 2, 500 mL) used in the analytical analyses. Blank volume of 500mL was assumed in the calculations...12

Table S8. Concentration (pg L^{-1}) of detected isomers in seawater samples. ∑PFAS consider all compounds above method detection limit (MDL). PFDS was always <MDL. 13

Table S9. Concentration (pg L^{-1}) of detected isomers in seawater samples. ∑PFAS consider all compounds above method detection limit (MDL). PFDS was always <MDL. 15

Figures

Figure S1. Temperature-salinity (T-S) diagram of the sampled transect from 15ºN to 23ºS. The colors indicate oxygen concentration in µmol kg-1 (scale at right). Density isopleths appears as grey line. TW: Tropical Water; NACW: North Atlantic Central Water; SACW: South Atlantic Central Water; AAIW: Antarctic Intermediate Water; NADW: North Atlantic Deep Water; AABW: Abyssal Antarctic Bottom Water. 16

Figure S2. Sampling points from six different studies: present study, Benskin et al. [20], González-Gaya et al. [11], Ahrens et al. [19], Ahrens et al. [18], and Zhao et al. [14]. 17

Figure S3. Backward air trajectories (72 hour) computed for sampling point #15.1 using NOAA's HYSPLIT Model. 18

Figure S4. Backward air trajectories (72 hour) computed for sampling point #16.1 using NOAA's HYSPLIT Model. 19

Figure S5. Vertical profiles of PFAS (pg L^{-1}) detected above MDL in ocean water columns from Tropical Atlantic Ocean (#1 (a) and #5 (b)) together with Salinity and Temperature (°C)................20

Figure S6. Vertical profiles of PFAS (pg L^{-1}) detected above MDL in ocean water columns from Tropical Atlantic Ocean (#7 (a) and #9 (b)) together with Salinity and Temperature (°C)................21

Figure S7. Vertical profile for conservative temperature, absolute salinity, oxygen, and dissolved oxygen for the sampled profile at 23ºS (#15). Black dots represent the depths where seawater samples were collected. 22

Figure S8. Vertical profile for conservative temperature, absolute salinity, oxygen, and dissolved oxygen for the sampled profile at 23ºS (#16). Black dots represent the depths where seawater samples were collected. 23

Figure S9. Vertical profiles of PFAS (pg L^{-1}) detected above MDL in ocean water columns from Rio de Janeiro upwelling (#15 (a) and #16 (b)) together with Salinity and Temperature (°C). 24

Standard and reagent brands

Methanol MeOH (HPLC grade) was purchased from J.T. Baker (Atlantic Labo, France) and Merck (Darmstadt, Germany). Acetonitrile was purchased from Honeywell (Steinheim, Germany). Formic acid was purchased from Merck (Darmstadt, Germany). Ammonium hydroxide salts was purchased from Mallinckrodt chemicals (Dublin, Ireland). All standards were purchased from Wellington Laboratories (Guelph, ON, Canada). Waters Oasis® Weak-anion exchange SPE cartridges (6 cm3, 150 mg, 30 µm) (Massachusetts, USA). SPE C18 cartridges were purchased from Phenomenex (California, USA). Lastly, water was purified with a Millipore water purification system (MilliQ, Massachusetts, USA) and had a resistance of 18.2 MΩ cm^{-1} for both Brazilian and Swedish laboratories.

PFAS-extraction method optimization

Prior to real samples analysis, PFAS were investigated in all WAX SPE cartridge batches, ultrapure water, reagents, and glassware in order to track contamination. There was only observed a systematic PFOA background in ultrapure water (~20 pg L^{-1}), which was associated with the Swedish tap water, as was seen before [1], besides other compounds in lower concentrations. To minimize the PFOA input in real samples, all ultrapure water was extracted following the same steps as this for real samples. However, PFHxA, PFOA, and PFBS were still been detected in the procedure blank tests at concentrations of 16;17±4; 18 pg L^{-1} (n = 3) after extraction, respectively. Then, the last step to eliminate possible contamination sources was the replacement of an in-use formic acid to a new lot of this reagent (sealed) resulting in laboratory blanks with PFAS concentrations lower than 1 pg L^{-1} (Table S7).

Table S1. Sample location, depth, temperature, salinity, oxygen and date of sampling. Pot. Temp.: Potential Temperature; Sal.: Salinity; Oxyg.: Oxygen; TW: Tropical Water; SACW: South Atlantic Central Water; AAIW: Antarctic Intermediate Water; mAAIW: modified AAIW; NADW: North Atlantic Deep Water; AABW: Antarctic Bottom Water.

Sample	Cruise Station	Latitude	Longitude	Depth (m)	Station Maximun Depth (m)	Pont. Temp. (°C)	Salinity	Oxyg (µmol/kg)	Neutral Density (kg/m^3)	Water masses	Date
**1.1**	PIRA_004	14°56'26.40"N	38° 2'44.40"W	8	5897	26.9	36.2	192	23.4	TW	2017-Nov
**1.2**		14°56'26.40"N	38° 2'44.40"W	82		22.4	36.8	189	25.5	TW	2017-Nov
**1.3**		14°56'26.40"N	38° 2'44.40"W	132		16.9	36.3	123	26.6	NACW	2017-Nov
**1.4**		14°56'26.40"N	38° 2'44.40"W	420		9.9	35.1	85	27.1	NACW	2017-Nov
**1.5**		14°56'26.40"N	38° 2'44.40"W	525		9.02	35.0	82	27.2	NACW	2017-Nov
**1.6**		14°56'26.40"N	38° 2'44.40"W	900		6.17	34.8	113	27.5	mAAIW	2017-Nov
**1.7**		14°56'26.40"N	38° 2'44.40"W	4501		1.93	34.9	233	28.1	NADW	2017-Nov
**1.8**		14°56'26.40"N	38° 2'44.40"W	5845		1.75	34.9	237	28.1	NADW	2017-Nov
**2.1**	PIRA_007	11°59'53.40"N	38° 6'49.80"W	5	4754	28.0	34.7	192	22.2	TW	2017-Nov
**3.1**	PIRA_011	7°59'13.80"N	38° 1'44.40"W	7	4221	27.9	35.9	191	23.1	TW	2017-Nov
**4.1**	PIRA_015	3°59'44.88"N	37°57'42.54"W	14	4236	28.1	36.0	190	23.1	TW	2017-Nov
**5.1**	PIRA_019	0° 2'41.76"N	38° 0'5.64"W	7	4448	27.2	36.2	191	23.4	TW	2017-Nov
**5.2**		0° 2'41.76"N	38° 0'5.64"W	135		20.7	36.5	179	25.7	SACW	2017-Nov
**5.3**		0° 2'41.76"N	38° 0'5.64"W	148		16.2	35.7	160	26.3	SACW	2017-Nov
**5.4**		0° 2'41.76"N	38° 0'5.64"W	376		9.39	34.8	138	27.0	SACW	2017-Nov
**5.5**		0° 2'41.76"N	38° 0'5.64"W	721		5.31	34.5	140	27.4	AAIW	2017-Nov
**5.6**		0° 2'41.76"N	38° 0'5.64"W	1780		3.78	35.0	234	27.9	NADW	2017-Nov
**5.7**		0° 2'41.76"N	38° 0'5.64"W	2901		2.55	34.9	245	28.1	NADW	2017-Nov
**5.8**		0° 2'41.76"N	38° 0'5.64"W	3844		1.96	34.9	249	28.1	NADW	2017-Nov
**5.9**		0° 2'41.76"N	38° 0'5.64"W	4431		0.75	34.7	217	28.2	AABW	2017-Nov
**6.1**	PIRA_021	1°59'17.28"S	38° 0'15.00"W	7	3166	27.2	36.3	192	23.7	TW	2017-Nov
**14.1**	PIRA_032	4°59'56.46"S	30° 0'1.86"W	6	5011	27.3	36.4	191	23.7	TW	2017-Dec
**7.1**	PIRA_035	8° 0'38.16"S	30°37'36.72"W	6	5402	27.3	36.5	190	23.6	TW	2017-Dec
**7.2**		8° 0'38.16"S	30°37'36.72"W	113		23.8	36.7	184	25.0	TW	2017-Dec
**7.3**		8° 0'38.16"S	30°37'36.72"W	152		18.5	36.1	133	26.0	SACW	2017-Dec
**7.4**		8° 0'38.16"S	30°37'36.72"W	375		9.11	34.8	103	27.0	SACW	2017-Dec
**7.5**		8° 0'38.16"S	30°37'36.72"W	473		7.98	34.7	83	27.1	SACW	2017-Dec
**7.6**		8° 0'38.16"S	30°37'36.72"W	833		4.54	34.5	150	27.5	AAIW	2017-Dec
**7.7**		8° 0'38.16"S	30°37'36.72"W	3579		2.11	34.9	242	28.1	NADW	2017-Dec
**7.8**		8° 0'38.16"S	30°37'36.72"W	5384		0.21	34.7	210	28.2	AABW	2017-Dec

Continuation of Table S1:

Sample	Cruise Station	Latitude	Longitude	Depth (m)	Station Maximun Depth (m)	Temperature (°C)	Salinity	Oxygen (µmol/kg)	Neutral Density (kg/m)	Water masses	Date
**8.1**	PIRA_053	13°59'18.60"S	32°46'13.80"W	7	4593	27.6	37.0	191	24.0	TW	2017-Dec
**9.1**	PIRA_058	18°50'54.84"S	34°40'40.80"W	6	4208	26.8	37.2	196	24.4	TW	2018-Jan
**9.2**		18°50'54.84"S	34°40'40.80"W	150		22.3	37.0	204	25.7	SACW	2018-Jan
**9.3**		18°50'54.84"S	34°40'40.80"W	551		7.79	34.6	167	27.1	SACW	2018-Jan
**9.4**		18°50'54.84"S	34°40'40.80"W	755		4.55	34.4	186	27.4	AAIW	2018-Jan
**9.5**		18°50'54.84"S	34°40'40.80"W	1400		4.07	34.9	204	27.8	NADW	2018-Jan
**9.6**		18°50'54.84"S	34°40'40.80"W	2501		2.90	34.9	240	28.0	NADW	2018-Jan
**9.7**		18°50'54.84"S	34°40'40.80"W	3800		1.54	34.8	231	28.1	NADW	2018-Jan
**9.8**		18°50'54.84"S	34°40'40.80"W	4194		0.50	34.7	214	28.2	AABW	2018-Jan
**10.1**	PIRA_060	21° 0'5.34"S	36°59'50.88"W	6	3996	27.3	37.2	196	24.3	TW	2018-Jan
**11.1**	PIRA_061	22° 0'2.76"S	38°30'1.68"W	6	3360	26.9	37.0	195	24.3	TW	2018-Jan
**12.1**	PIRA_062	22°59'57.00"S	39°59'54.00"W	7	2706	26.9	37.2	195	24.4	TW	2018-Jan
**13.1**	PIRA_063	23° 3'1.80"S	40°53'58.80"W	5	2691	25.5	36.9	199	24.6	TW	2018-Jan
**15.1**	PIRA_064	23° 2'11.40"S	41°38'4.20"W	7	83	23.8	36.0	211	24.4	TW	2018-Jan
**15.2**		23° 2'11.40"S	41°38'4.20"W	49		19.6	36.4	191	25.9	SACW	2018-Jan
**15.3**		23° 2'11.40"S	41°38'4.20"W	65		18.0	36.0	177	26.0	SACW	2018-Jan
**15.4**		23° 2'11.40"S	41°38'4.20"W	75		14.7	35.5	170	26.5	SACW	2018-Jan
**16.1**	PIRA_065	23° 3'23.40"S	42°25'9.60"W	7	75	20.0	36.1	209	25.7	SACW	2018-Jan
**16.2**		23° 3'23.40"S	42°25'9.60"W	21		18.1	36.0	225	26.0	SACW	2018-Jan
**16.3**		23° 3'23.40"S	42°25'9.60"W	32		16.5	35.8	183	26.3	SACW	2018-Jan
**16.4**		23° 3'23.40"S	42°25'9.60"W	68		13.7	35.3	191	26.6	SACW	2018-Jan

Table S2. Water masses characteristics. Where = TW: Tropical Water; NACW: North Atlantic Central Water; SACW: South Atlantic Central Water; AAIW: Antarctic Intermediate Water; mAAIW: Mediterranean AAIW; NADW: North Atlantic Deep Water; AABW: Abyssal Antarctic Bottom Water.

Water masses	Depth (m)	Temperature (°C)	Salinity	Neutral Density (Kg/m³)
**TW**	0 – 116[a]	> 20[c]	> 36[c]	< 25.7[d]
**NACW**	100 – 700[a]	6-18[b]	34.5 – 36[b]	26 – 27.2[b]
**SACW**	116 – 657[a]	6 – 20[d]	34.3 – 36[a]	25.7 – 27.2 [a]
**AAIW**	657 – 1234[i]	3 - -6[e]	34.2 - 34.6[e]	27.4 - 27.8[a]
**mAAIW**	657 – 1234[g]	5.6 -6.5[g]	34.7 – 34.9[g]	27.4 – 27.8[g]
**NADW**	1234 – 3472[h]	3 - -4[f]	34.6 – 35[f]	27.8 – 28.2[h]
**AABW**	> 3472[i]	-1.9[h]	34.6[h]	> 28.2[h]

Based on: [a]Stramma and England [2]; [b]Liu & Tanhua [3]; [c]Emilsson [4]; [d]Miranda et al. [5]; [e]Sverdrup et al. [6]; [f]Silveira [7]; [g]Bashmachnikov et al. [8]; [h]Ferreira & Kerr [9]; [i]Talley [10].

Table S3. Perfluoroalkyl substances (PFAS) analyzed in the present study.

Acronym	Name	Formula	CAS#
	**Perfluoroalkyl carboxylic acids (PFCAs)**		
PFHxA	Perfluorohexanoic acid	$C_5F_{11}COOH$	307-24-4
PFHpA	Perfluoroheptanoic acid	$C_6F_{13}COOH$	375-85-9
PFOA*	Perfluorooctanoic acid	$C_7F_{15}COOH$	335-67-1
PFNA	Perfluorononanoic acid	$C_8F_{17}COOH$	375-95-1
PFDA	Perfluorodecanoic acid	$C_9F_{19}COOH$	335-76-2
PFUnDA	Perfluoroundecanoic acid	$C_{10}F_{21}COOH$	2058-94-8
PFDoDA	Perfluorododecanoic acid	$C_{11}F_{23}COOH$	307-55-1
PFTriDA	Perfluorotridecanoic acid	$C_{12}F_{25}COOH$	72629-94-8
PFTeDA	Perfluorotetradecanoic acid	$C_{13}F_{27}COOH$	376-06-7
	**Perfluoroalkyl sulfonic acids (PFSAs)**		
PFBS	Perfluorobutanesulfonic acid	$C_4F_9SO_3H$	375-73-5
PFHxS*	Perfluorohexanesulfonic acid	$C_6F_{13}SO_3H$	355-46-4
PFOS*	Perfluorooctanesulfonic acid	$C_8F_{17}SO_3H$	1763-23-1
PFDS*	Perfluorodecanesulfonic acid	$C_{10}F_{21}SO_3H$	355-77-3
	**Perfluoroalkyl sulfonamide derivatives (FASAs)**		
FOSA*	Perfluorooctanesulfonamide	$C_8F_{17}SO_2NH_2$	754-91-6

*Compounds analysed for both linear (L-) and branched (Br-) isomers.

Table S4. Mobile phase gradient profile used in LC-MS/MS.

	**LC Gradient Program**		**LC Flow Rate**
Time (min)	Mobile phase A (%)[1]	Mobile Phase B (%)[2]	(mL/min)
0.0	90	10	0.40
0.3	90	10	0.40
4.5	20	80	0.40
4.6	0	100	0.40
7.5	0	100	0.55
9.5	90	10	0.40

[1] Mobile phase A: 90 % water and 10 % acetonitrile containing 2 mM ammonium acetate.
[2] Mobile phase B: 99 % acetonitrile and 1% water containing 2 mM ammonium acetate.

Table S5. List of retention times and monitored qualitative and quantitative ions for each compound analyzed in the present study.

Target	Retention Time (min)	Quant. Ion (m/z)	Qual Ion (m/z)	Standard	Internal Standard	IS Ion	Data quality
L-PFHxA	2.49	313/269	313/119	L-PFHxA	^{13}C-PFHxA	315/270	Quantitative
L-PFHpA	2.86	363/319	363/169	L-PFHpA	^{13}C-PFHpA	367/322	Quantitative
L-PFOA	3.19	413/369	413/169	L-PFOA	^{13}C-PFOA	417/372	Quantitative
Br-PFOA	3.15	413/369	413/169	L-PFOA	^{13}C-PFOA	417/372	Semi-quantitative
L-PFNA	3.43	463/419	463/219	L-PFNA	^{13}C-PFNA	468/423	Quantitative
L-PFDA	3.22	513/469	513/269	L-PFDA	^{13}C-PFDA	515/470	Quantitative
L-PFUnDA	3.97	563/519	563/269	L-PFUnDA	^{13}C-PFUnDA	565/520	Quantitative
L-PFDoDA	4.23	613/569	613/169	L-PFDoDA	^{13}C-PFDoA	615/570	Quantitative
L-PFTrDA	4.51	663/619	663/169	L-PFTrDA	^{13}C-PFDoA	615/570	Quantitative
L-PFTeDA	3.97	713/669	713/169	L-PFTeDA	^{13}C-PFDoA	615/570	Quantitative
L-PFBS	2.45	299/80	299/99	L-PFBS	^{18}O-PFHxS	403/84	Quantitative
L-PFHxS	3.25	399/80	399/99	L-PFHxS	^{18}O-PFHxS	403/84	Quantitative
Br-PFHxS	~3.22	399/80	399/99	L-PFHxS	^{18}O-PFHxS	403/84	Semi-quantitative
L-PFOS	3.84	499/80	499/99	L-PFOS	^{13}C-PFOS	503/80	Quantitative
Br-PFOS	~3.81	499/80	499/99	L-PFOS	^{13}C--PFOS	503/80	Semi-quantitative
L-PFDS	4.34	599/80	599/99	L-PFDS	^{13}C-PFOS	503/80	Quantitative
Br-PFDS	~4.31	599/80	599/99	L-PFDS	^{13}C-PFOS	503/80	Semi-quantitative
L-FOSA	4.87	498/78	498/169	L-FOSA	^{13}C-FOSA	506/78	Quantitative
Br-FOSA	~4.84	498/78	498/169	L-FOSA	^{13}C-FOSA	506/78	Semi-quantitative

Table S6. Values for method detection limit (MDL), method quantification limit (MQL), and spike recovery used in the analytical analyses (n = 8, each). It is based on 500 mL extractions. Branched isomers MDL and MQL are the same as their linear isomers.

	MDL	MQL	Spike/recovery experiments			
			50 pg		500 pg	
	pg L^{-1}	pg L^{-1}	Average (%)	RSD (%)	Average (%)	RSD (%)
PFHxA	1.92	6.40	78	9	100	6
PFHpA	5.30	17.7	76	18	98	11
L-PFOA	2.98	9.92	78	14	103	12
PFNA	1.71	5.69	78	14	100	9
PFDA	0.50	1.67	75	11	100	10
PFUnDA	1.85	6.18	87	23	90	17
PFDoDA	0.50	1.67	83	15	90	32
PFTriDA	6.88	22.9	40	12	61	21
PFTeDA	0.50	1.67	27	8	34	11
PFBS	2.88	9.60	124	64	136	124
L-PFHxS	1.59	5.30	70	18	88	10
L-PFOS	0.59	1.98	92	23	110	47
L-PFDS	3.93	13.1	51	22	65	22
L-FOSA	0.89	2.97	81	27	132	84

Table S7. Values for field (n = 3, 1 mL), lab (n = 7, 1 mL) bottle (n = 2, 500 mL) used in the analytical analyses. Blank volume of 500 mL was assumed in the calculations.

	Brazil			Sweden			Bottle		
**Compound**	**Avg (pg L^{-1})**	**SD**	**Con. in 1 mL**	**Avg (pg L^{-1})**	**SD**	**Con. in 1 mL**	**Avg (pg L^{-1})**	**SD**	**Con. in 1 mL**
PFHxA	5.97	5.3	< 0.0060	< 1.92		< 0.0019	< 1.92		< 0.0019
PFHpA	< 5.30		< 0.0053	< 5.30		< 0.0053	< 5.30		< 0.0053
L-PFOA	<2.98		< 0.0030	<2.98		< 0.0030	<2.98		< 0.0030
Br-PFOA	<2.98		< 0.0030	<2.98		< 0.0030	<2.98		< 0.0030
PFNA	< 1.71		< 0.0017	< 1.71		< 0.0017	< 1.71		< 0.0017
PFDA	1.12	1.6	< 0.0011	0.70	1.5	< 0.0007	< 0.50		< 0.0005
PFUnDA	< 1.85		< 0.0019	< 1.85		< 0.0019	< 1.85		< 0.0019
PFDoDA	< 0.50		< 0.0005	< 0.50		< 0.0005	0.66	0.7	< 0.0007
PFTriDA	< 6.88		< 0.0069	< 6.88		< 0.0069	< 6.88		< 0.0069
PFTeDA	< 0.50		< 0.0005	< 0.50		< 0.0005	< 0.50		< 0.0005
PFBS	< 2.88		< 0.0029	< 2.88		< 0.0029	< 2.88		< 0.0029
L-PFHxS	< 1.59		< 0.0016	< 1.59		< 0.0016	< 1.59		< 0.0016
Br-PFHxS	< 1.59		< 0.0016	< 1.59		< 0.0016	< 1.59		< 0.0016
L-PFOS	4.55	6.4	< 0.0045	< 0.59		< 0.0006	< 0.59		< 0.0006
Br-PFOS	< 0.59		< 0.0006	< 0.59		< 0.0006	< 0.59		< 0.0006
L-PFDS	< 3.93		< 0.0039	< 3.93		< 0.0039	< 3.93		< 0.0039
Br-PFDS	< 3.93		< 0.0039	< 3.93		< 0.0039	< 3.93		< 0.0039
L-FOSA	< 0.89		< 0.0009	< 0.89		< 0.0009	< 0.89		< 0.0009
Br-FOSA	< 0.89		< 0.0009	< 0.89		< 0.0009	< 0.89		< 0.0009

[1]Lab blanks of PFHxA, L-PFOA, and PFHxS n = 7 due to the first batch with contamination of reagents for these compounds (5.18, 68.5 and 23.0 pg L^{-1}, respectively).

Table S8. Concentration (pg L^{-1}) of detected isomers in seawater samples. ∑PFAS consider all compounds above method detection limit (MDL). PFDS was always <MDL.

**Sample n.**	**Water masses**	**L-PFHxA**	**Br-PFHxA**	**PFHpA**	**L-PFOA**	**Br-PFOA**	**PFNA**	**PFDA**	**PFUnDA**	**PFDoDA**	**PFTriDA**	**PFTeDA**	**PFBS**	**L-PFHxS**	**L-PFOS**	**Br-PFOS**	**L-FOSA**	**Br-FOSA**	**∑ PFAA**
**1.1**	TW	n.r.	n.r.	19.3	n.r.	n.r.	(4.86)	1.78	(1.85)	10.5	< 6.88	< 0.50	< 2.88	n.r.	< 0.59	< 0.59	< 0.89	< 0.89	38.3
**1.2**	TW	n.r.	n.r.	(16.0)	n.r.	n.r.	(10.8)	(1.56)	10.7	< 0.50	< 6.88	(0.86)	< 2.88	n.r.	2.92	< 0.59	< 0.89	< 0.89	42.8
**1.3**	NACW	n.r.	n.r.	24.3	n.r.	n.r.	(17.0)	< 0.50	(2.47)	< 0.50	< 6.88	< 0.50	< 2.88	n.r.	10.1	< 0.59	(1.91)	< 0.89	55.8
**1.4**	NACW	n.r.	n.r.	20.0	n.r.	n.r.	(2.39)	< 0.50	< 1.85	< 0.50	< 6.88	< 0.50	< 2.88	n.r.	1.97	< 0.59	5.20	< 0.89	29.6
**1.5**	NACW	n.r.	n.r.	60.5	n.r.	n.r.	(1.86)	< 0.50	< 1.85	< 0.50	< 6.88	< 0.50	< 2.88	n.r.	< 0.59	< 0.59	3.19	< 0.89	65.6
**1.6**	mAAIW	n.r.	n.r.	50.1	n.r.	n.r.	< 1.71	(1.56)	15.0	< 0.50	< 6.88	10.5	< 2.88	n.r.	< 0.59	< 0.59	< 0.89	< 0.89	77.2
**1.7**	NADW	n.r.	n.r.	< 5.30	n.r.	n.r.	(2.02)	< 0.50	4.86	< 0.50	< 6.88	< 0.50	< 2.88	n.r.	< 0.59	< 0.59	4.66	< 0.89	11.5
**1.8**	NADW	n.r.	n.r.	< 5.30	n.r.	n.r.	< 1.71	(1.22)	< 1.85	< 0.50	< 6.88	< 0.50	< 2.88	n.r.	< 0.59	< 0.59	< 0.89	< 0.89	1.22
**2.1**	Surface	7.16	< 1.92	< 5.30	< 2.98	< 2.99	(3.91)	< 0.50	< 1.85	< 0.50	< 6.88	< 0.50	< 2.88	< 1.59	< 0.59	< 0.59	< 0.89	< 0.89	11.1
**3.1**	Surface	8.63	< 1.92	(6.00)	(6.11)	< 2.98	7.47	< 0.50	< 1.85	< 0.50	< 6.88	< 0.50	< 2.88	< 1.59	< 0.59	< 0.59	< 0.89	< 0.89	28.2
**4.1**	Surface	< 1.92	< 1.92	(11.9)	(6.81)	(3.48)	< 1.71	< 0.50	< 1.85	< 0.50	< 6.88	< 0.50	< 2.88	< 1.59	< 0.59	< 0.59	< 0.89	< 0.89	22.2
**5.1**	TW	< 1.92	< 1.92	< 5.30	< 2.98	< 2.98	< 1.71	< 0.50	< 1.85	< 0.50	< 6.88	< 0.50	< 2.88	< 1.59	< 0.59	< 0.59	< 0.89	< 0.89	n.d.
**5.2**	SACW	< 1.92	< 1.92	< 5.30	< 2.98	< 2.98	(2.67)	< 0.50	< 1.85	< 0.50	< 6.88	< 0.50	< 2.88	< 1.59	< 0.59	< 0.59	11.8	< 0.89	14.5
**5.3**	SACW	< 1.92	< 1.92	< 5.30	< 2.98	< 2.98	< 1.71	16.6	< 1.85	< 0.50	< 6.88	< 0.50	< 2.88	< 1.59	< 0.59	< 0.59	< 0.89	< 0.89	16.6
**5.4**	SACW	< 1.92	< 1.92	< 5.30	29.6	< 2.98	< 1.71	< 0.50	< 1.85	< 0.50	< 6.88	< 0.50	< 2.88	< 1.59	< 0.59	< 0.59	106	< 0.89	136
**5.5**	AAIW	< 1.92	< 1.92	< 5.30	< 2.98	< 2.98	< 1.71	< 0.50	< 1.85	< 0.50	< 6.88	< 0.50	< 2.88	< 1.59	< 0.59	< 0.59	< 0.89	< 0.89	n.d.
**5.6**	NADW	< 1.92	< 1.92	< 5.30	< 2.98	< 2.98	< 1.71	< 0.50	< 1.85	< 0.50	< 6.88	< 0.50	< 2.88	< 1.59	< 0.59	< 0.59	< 0.89	< 0.89	n.d.
**5.7**	NADW	< 1.92	< 1.92	< 5.30	< 2.98	< 2.98	< 1.71	< 0.50	< 1.85	< 0.50	< 6.88	< 0.50	< 2.88	< 1.59	< 0.59	< 0.59	< 0.89	< 0.89	n.d.
**5.8**	NADW	< 1.92	< 1.92	< 5.30	(3.03)	< 2.98	< 1.71	< 0.50	< 1.85	< 0.50	< 6.88	< 0.50	< 2.88	< 1.59	< 0.59	< 0.59	< 0.89	< 0.89	3.03
**5.9**	AABW	< 1.92	< 1.92	< 5.30	28.9	< 2.98	< 1.71	< 0.50	< 1.85	< 0.50	< 6.88	< 0.50	< 2.88	< 1.59	< 0.59	< 0.59	< 0.89	< 0.89	28.9
**6.1**	TW	< 1.92	< 1.92	< 5.30	(9.78)	(1.82)	(1.93)	< 0.50	< 1.85	< 0.50	< 6.88	< 0.50	< 2.88	< 1.59	< 0.59	< 0.59	< 0.89	< 0.89	13.5
**14.1**	TW	11.8	< 1.92	(6.50)	(9.74)	< 2.98	< 1.71	1.78	< 1.85	5.30	< 6.88	< 0.50	< 2.88	< 1.59	< 0.59	< 0.59	< 0.89	< 0.89	35.1
**7.1**	TW	6.15	< 1.92	< 5.30	19.7	11.0	< 1.71	< 0.50	< 1.85	< 0.50	< 6.88	< 0.50	< 2.88	< 1.59	< 0.59	< 0.59	9.82	< 0.89	46.7
**7.2**	TW	< 1.92	< 1.92	(11.2)	10.7	< 2.98	(5.40)	(0.98)	< 1.85	< 0.50	< 6.88	< 0.50	< 2.88	< 1.59	< 0.59	< 0.59	(2.42)	< 0.89	30.7
**7.3**	SACW	< 1.92	< 1.92	(15.8)	9.98	< 2.98	< 1.71	< 0.50	< 1.85	< 0.50	< 6.88	2.60	< 2.88	< 1.59	< 0.59	< 0.59	(1.23)	< 0.89	29.6
**7.4**	SACW	< 1.92	< 1.92	80.2	(9.29)	< 2.98	< 1.71	(1.07)	(3.93)	< 0.50	< 6.88	< 0.50	< 2.88	< 1.59	< 0.59	< 0.59	64.5	< 0.89	159
**7.5**	SACW	< 1.92	< 1.92	< 5.30	20.9	13.0	< 1.71	(0.95)	< 1.85	< 0.50	< 6.88	< 0.50	< 2.88	< 1.59	< 0.59	< 0.59	25.1	< 0.89	60.0
**7.6**	AAIW	< 1.92	< 1.92	< 5.30	< 2.98	< 2.98	< 1.71	<0.50	< 1.85	2.85	< 6.88	< 0.50	< 2.88	< 1.59	< 0.59	< 0.59	< 0.89	< 0.89	2.85
**7.7**	NADW	< 1.92	< 1.92	(12.7)	< 2.98	< 2.98	< 1.71	< 0.50	< 1.85	< 0.50	< 6.88	< 0.50	< 2.88	< 1.59	< 0.59	< 0.59	8.07	< 0.89	20.8
**7.8**	AABW	< 1.92	< 1.92	< 5.30	< 2.98	< 2.98	< 1.71	2.63	< 1.85	< 0.50	< 6.88	< 0.50	< 2.88	< 1.59	< 0.59	< 0.59	25.9	< 0.89	28.5

n.r. = values non reported due to analytical issues.
Numbers in brackets are above method detection limit (MDL) but below the respective MQL. <x: below the respective MDL

Continuation of Table S8:

**Sample n.**	**Water masses**	**L-PFHxA**	**Br-PFHxA**	**PFHpA**	**L-PFOA**	**Br-PFOA**	**PFNA**	**PFDA**	**PFUnDA**	**PFDoDA**	**PFTriDA**	**PFTeDA**	**PFBS**	**L-PFHxS**	**L-PFOS**	**Br-PFOS**	**L-FOSA**	**Br-FOSA**	**∑ PFAA**
**8.1**	TW	9.58	< 1.92	(14.1)	(3.16)	< 2.98	< 1.71	2.59	< 1.85	< 0.50	< 6.88	< 0.50	< 2.88	< 1.59	< 0.59	< 0.59	< 0.89	< 0.89	29.4
**9.1**	TW	< 1.92	< 1.92	< 5.30	(9.25)	< 2.98	(5.68)	< 0.50	< 1.85	< 0.50	< 6.88	< 0.50	< 2.88	< 1.59	< 0.59	< 0.59	< 0.89	< 0.89	14.9
**9.2**	SACW	9.52	< 1.92	< 5.30	< 2.98	< 2.98	< 1.71	3.00	< 1.85	(1.37)	< 6.88	< 0.50	< 2.88	(3.24)	< 0.59	< 0.59	3.29	< 0.89	20.4
**9.3**	SACW	< 1.92	< 1.92	37.1	22.0	< 2.98	< 1.71	< 0.50	< 1.85	< 0.50	< 6.88	< 0.50	< 2.88	< 1.59	< 0.59	< 0.59	8.26	< 0.89	67.4
**9.4**	AAIW	< 1.92	< 1.92	20.0	10.8	< 2.98	< 1.71	2.78	< 1.85	< 0.50	< 6.88	< 0.50	< 2.88	< 1.59	< 0.59	< 0.59	(1.49)	< 0.89	35.1
**9.5**	NADW	< 1.92	< 1.92	47.9	15.4	(7.07)	< 1.71	(0.70)	< 1.85	< 0.50	< 6.88	< 0.50	< 2.88	< 1.59	1.44	< 0.59	8.27	< 0.89	80.8
**9.6**	NADW	< 1.92	< 1.92	73.6	14.4	(3.59)	< 1.71	< 0.50	< 1.85	< 0.50	< 6.88	< 0.50	< 2.88	< 1.59	< 0.59	< 0.59	< 0.89	< 0.89	91.6
**9.7**	NADW	< 1.92	< 1.92	< 5.30	16.5	(9.91)	(5.04)	< 0.50	< 1.85	< 0.50	< 6.88	< 0.50	< 2.88	< 1.59	< 0.59	< 0.59	< 0.89	< 0.89	31.5
**9.8**	AABW	< 1.92	< 1.92	< 5.30	(6.39)	< 2.98	< 1.71	< 0.50	(2.08)	< 0.50	< 6.88	< 0.50	< 2.88	< 1.59	< 0.59	< 0.59	< 0.89	< 0.89	8.47
**10.1**	TW	< 1.92	< 1.92	< 5.30	< 2.98	< 2.98	< 1.71	< 0.50	< 1.85	< 0.50	< 6.88	< 0.50	< 2.88	< 1.59	< 0.59	< 0.59	< 0.89	< 0.89	n.d.
**11.1**	TW	< 1.92	< 1.92	< 5.30	14.9	< 2.98	< 1.71	< 0.50	< 1.85	< 0.50	< 6.88	< 0.50	(7.35)	< 1.59	< 0.59	< 0.59	< 0.89	< 0.89	22.3
**12.1**	TW	< 1.92	< 1.92	22.9	< 2.98	< 2.98	< 1.71	< 0.50	< 1.85	< 0.50	< 6.88	< 0.50	< 2.88	< 1.59	< 0.59	< 0.59	< 0.89	< 0.89	22.9
**13.1**	TW	< 1.92	< 1.92	< 5.30	< 2.98	< 2.98	< 1.71	< 0.50	< 1.85	< 0.50	< 6.88	< 0.50	< 2.88	< 1.59	< 0.59	< 0.59	< 0.89	< 0.89	n.d.
**15.1**	TW	17.7	< 1.92	(16.0)	29.5	< 2.98	< 1.71	< 0.50	< 1.85	< 0.50	< 6.88	< 0.50	< 2.88	< 1.59	< 0.59	< 0.59	5.78	< 0.89	69.0
**15.2**	SACW	< 1.92	< 1.92	< 5.30	(5.62)	< 2.98	< 1.71	(1.12)	< 1.85	< 0.50	< 6.88	< 0.50	< 2.88	< 1.59	< 0.59	< 0.59	< 0.89	< 0.89	6.7
**15.3**	SACW	10.1	< 1.92	54.3	11.1	4.995	< 1.71	(1.18)	< 1.85	(1.41)	< 6.88	< 0.50	< 2.88	< 1.59	3.75	< 0.59	< 0.89	< 0.89	87
**15.4**	SACW	34.3	< 1.92	123	19.6	< 2.98	< 1.71	3.15	< 1.85	(0.67)	< 6.88	< 0.50	< 2.88	< 1.59	< 0.59	< 0.59	12.9	< 0.89	194
**16.1**	SACW	< 1.92	< 1.92	< 5.30	(4.69)	< 2.98	(3.10)	< 0.50	< 1.85	< 0.50	< 6.88	< 0.50	(4.02)	< 1.59	< 0.59	< 0.59	2.39	< 0.89	14.2
**16.2**	SACW	< 1.92	< 1.92	31.5	(18.3)	< 2.98	< 1.71	< 0.50	< 1.85	< 0.50	(7.52)	< 0.50	< 2.88	(2.30)	< 0.59	< 0.59	< 0.89	< 0.89	60
**16.3**	SACW	< 1.92	< 1.92	173	(8.36)	< 2.98	< 1.71	< 0.50	< 1.85	6.61	(9.40)	< 0.50	< 2.88	< 1.59	< 0.59	< 0.59	0.95	< 0.89	198
**16.4**	SACW	< 1.92	< 1.92	86.5	(9.00)	< 2.98	< 1.71	< 0.50	< 1.85	< 0.50	< 6.88	< 0.50	< 2.88	< 1.59	3.06	< 0.59	5.50	< 0.89	104

Table S9. Concentration (pg L^{-1}) of detected isomers in seawater samples. ∑PFAS consider all compounds above method detection limit (MDL). PFDS was always <MDL.

Reference	Sampling Year	Position	PFHxA	PFHpA	L-PFOA	PFNA	PFDA	PFDoDA	PFBS	L-PFHxS	L-PFOS	L-FOSA
**Atlantic**												
this study	2017	15°N-23°S	< 1.92 - 17.7.0	< 17.7 - 22.9	< 9.92 - 29.4	< 1.71 - 7.47	< 0.50 - 2.59	< 1.67 - 11.0	nd	nd	nd	< 2.97 - 9.82
González-Gaya et al. (2014)	2011	15°N-24°S	nd - 39	3.12 - 54	18.4 - 81.4	4.24 - 384	nd - 110	na	14.1 - 480	14.6 - 77.4	190 - 1100	nd - 6.00
Zhao et al. (2012)	2010	61°N-70°S	< 5.9 - 85	na	< 13 - 160	< 12 - 39	< 21	< 25	< 51 - 65	< 6.5 - 45	< 20 - 25	< 83
Benskin et al. (2012)	2005	11°N-21°S	14 -75	7 - 51	17 - 32	5.5 - 19	3.3 - 9.3	nd	nr	1.4 - 4.9	13 - 32	2 - 19
	2007	12°N-25°S	nd - 41	nd - 18	5.2 - 41	2.4 - 15	2.9 - 9.2	1.3 - 55	nd - 50	nd - 8.0	18 - 52	nd -2.7
Ahrens et al. (2009)	2008	15°N-26°S	< 5.7	< 5.9 - 9.7	< 4.0 - 87	< 5.1 - 35	na	na	< 1.6	nd	< 10 - 60	< 17 - 60
Ahrens et al. (2010)	2009	52°N-69°S	<3.0-117	<3.0-28	<5.2-223	<3.0-39	<5.5-37	<5.9-48	<4.4-50	<4.1-53	<11-232	<3.067
Yamashita et al. (2005)	2004	0°N-10°N	na	na	100 - 439	na	na	na	na	2.6 - 12	37 - 73	na
**Pacific**												
González-Gaya et al. (2014)	2011	35-0º N	nd	nd - 296	9 - 138	13 - 94	nd - 243	na	nd - 115	nd - 161	4 - 476	2 - 9
	2011	0 - 40°S	nd	nd - 143	3 - 164	16 - 35	nd - 87	na	nd - 110	nd - 71	2 - 13	nd - 6.00
**Indian**												
González-Gaya et al. (2014)	2011	0-40º S	nd	nd - 51	5 - 45	11 - 60	9 - 120	na	7 - 124	nd - 7	5 - 53	nd - 3
Wei et al. (2007)	2007	*	nd	nd	nd - 11.9	nd - 11	nd - 5.4	nd - 1.4	nd - 2.9	nd	nd - 23.9	na
**Arctic**		70°N	<1.0 - 31	<1.0 - 30	9.0 - 48.5	9 - 29.0	<1.0 - 9	<1.0 - 8	<1.0 - 18	<1.0 - 15	<1.0 - 41	na
Yeung et al. (2017)	2012	71°N	< 1.6 - 45									
Zhao et al. (2012)	2009	Greenland sea	< 5.9 - 38	na	< 13 - 160	< 12 - 16	< 21	< 25	< 51 - 65	< 6.5 - 45	< 20 - 25	< 83
**Antarctic**												
Cai et al. (2012)	2011	62°S	56.6 - 361	< 5.6 - 28.1	81.1 - 15,096	nd	nd	< 4.3 - 35.8	< 8.3 - 11.3	nd	nd	< 40.3 - 46.4
Wei et al. (2007)	2007	*	56.6 - 360	< 5.6 - 28.1	< 5.0	< 5.0	< 5.0	na	< 1.0 - 2.9	< 1.0	na	< 40.3 - 46.4
Zhao et al. (2012)	2010	63°S - 66°S	< 5.9	na	<13 - 15	< 12	< 21	< 25	< 51	< 6.5	< 20 - 46	< 83

* The coordinates of the sampling were not described by the author [11–19]

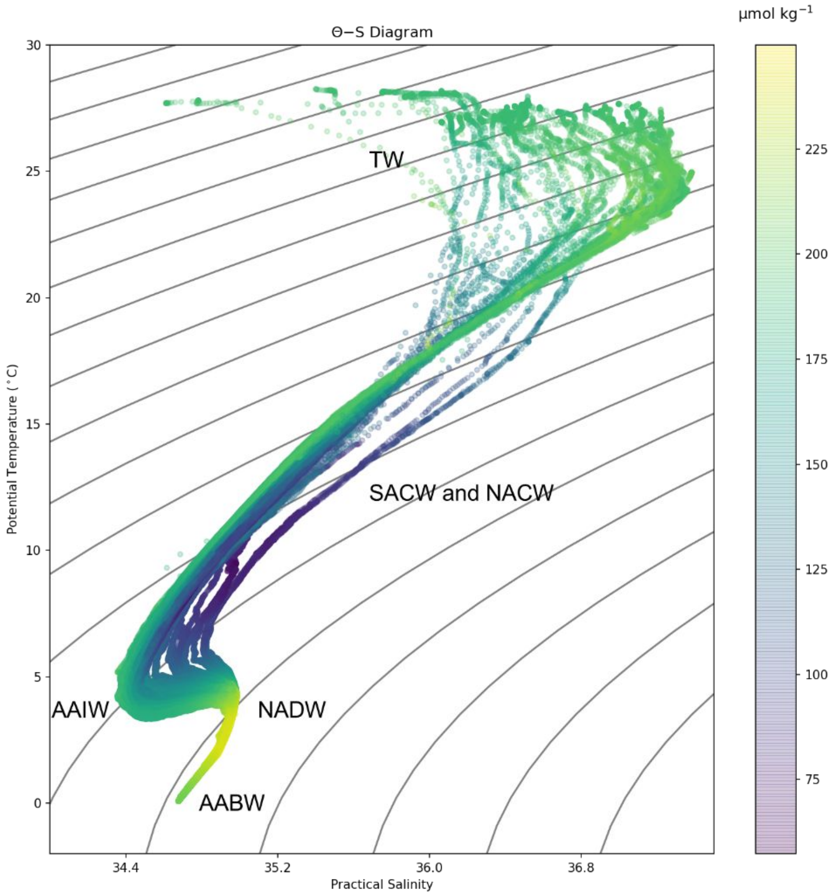

Figure S1. Temperature-salinity (T-S) diagram of the sampled transect from 15ºN to 23ºS. The colors indicate oxygen concentration in µmol kg-1 (scale at right). Density isopleths appears as grey line. TW: Tropical Water; NACW: North Atlantic Central Water; SACW: South Atlantic Central Water; AAIW: Antarctic Intermediate Water; NADW: North Atlantic Deep Water; AABW: Abyssal Antarctic Bottom Water.

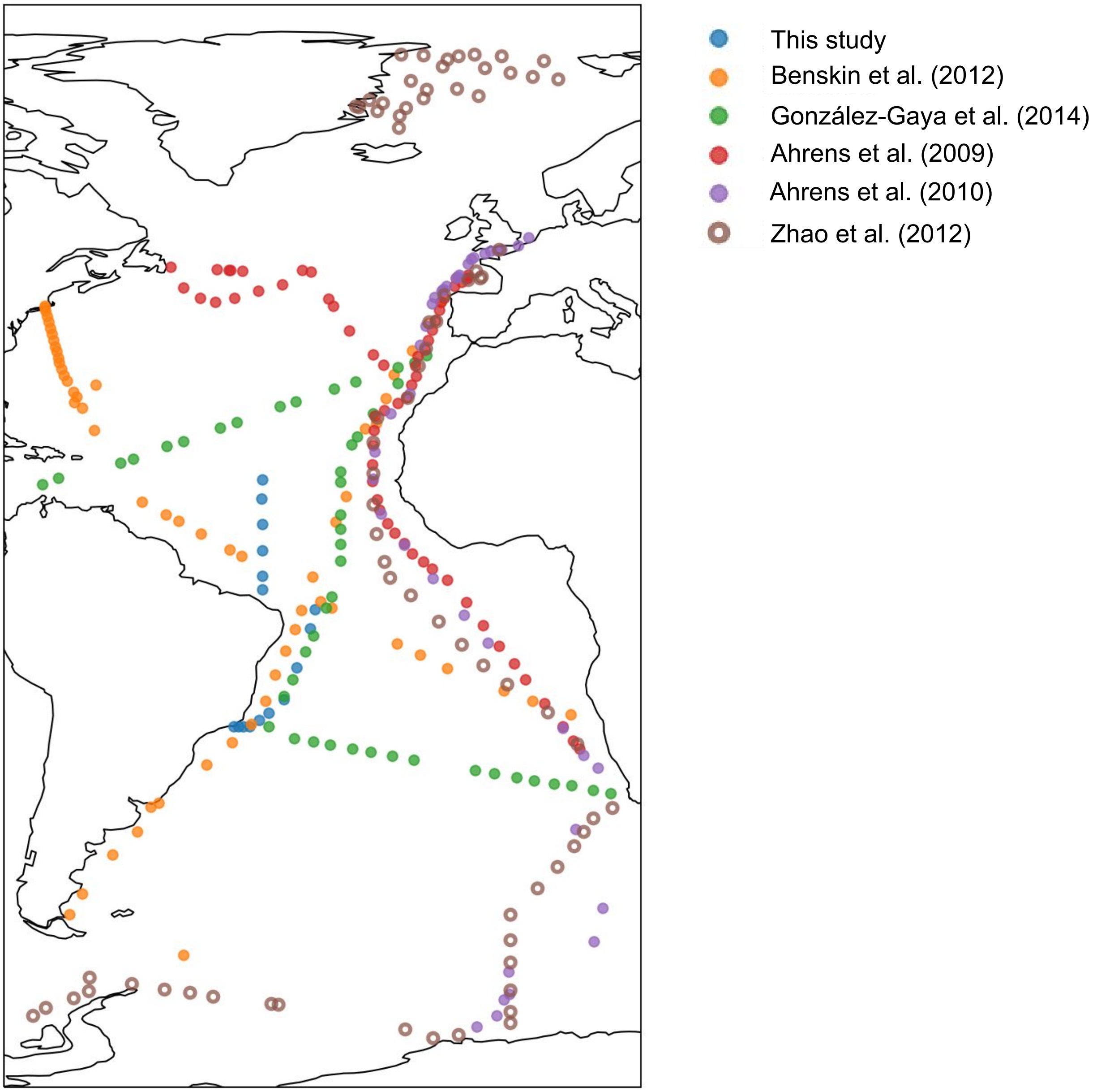

Figure S2. Sampling points from six different studies: present study, Benskin et al. [20], González-Gaya et al. [11], Ahrens et al. [19], Ahrens et al. [18], and Zhao et al. [14].

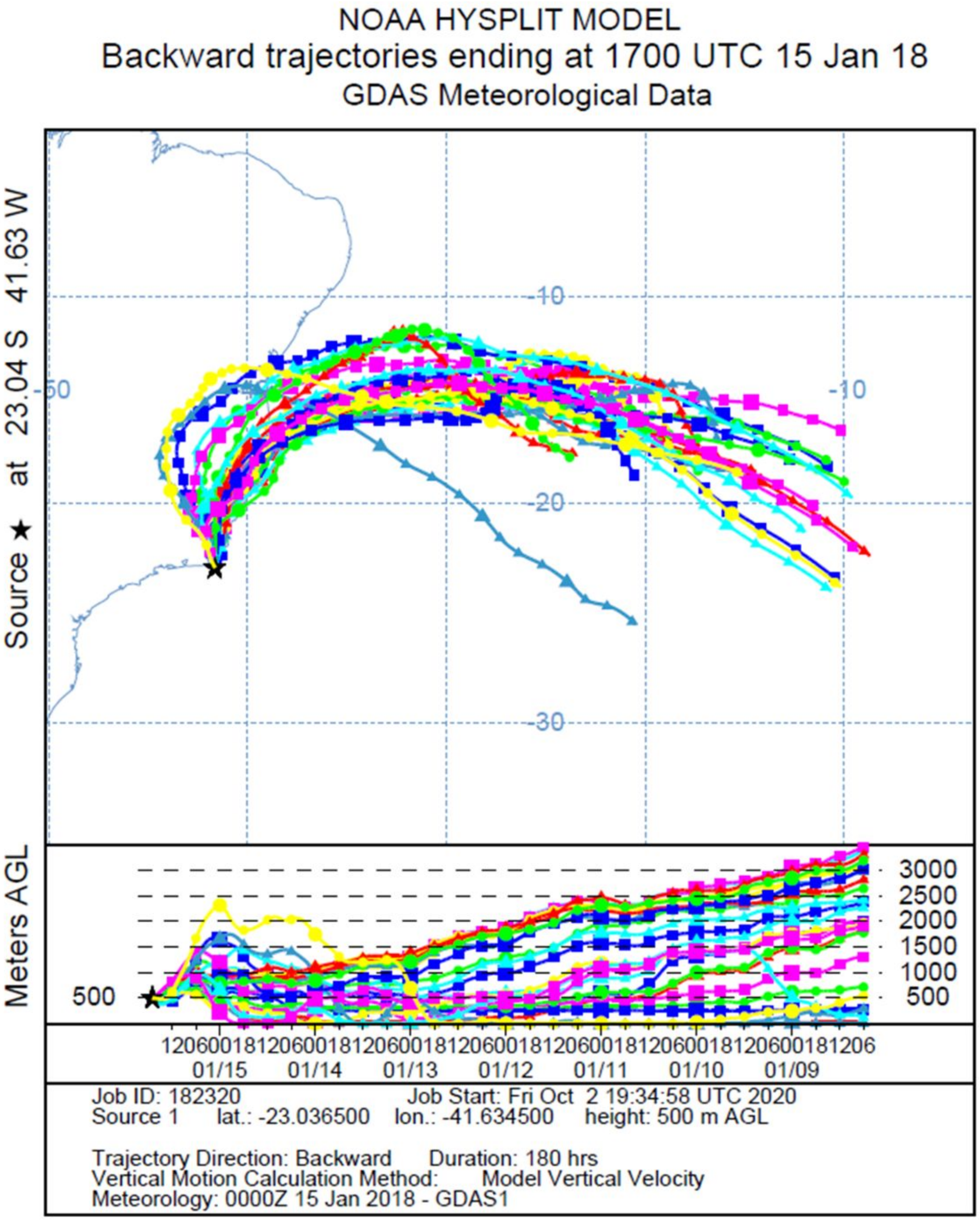

Figure S3. Backward air trajectories (72 hour) computed for sampling point #15.1 using NOAA's HYSPLIT Model.

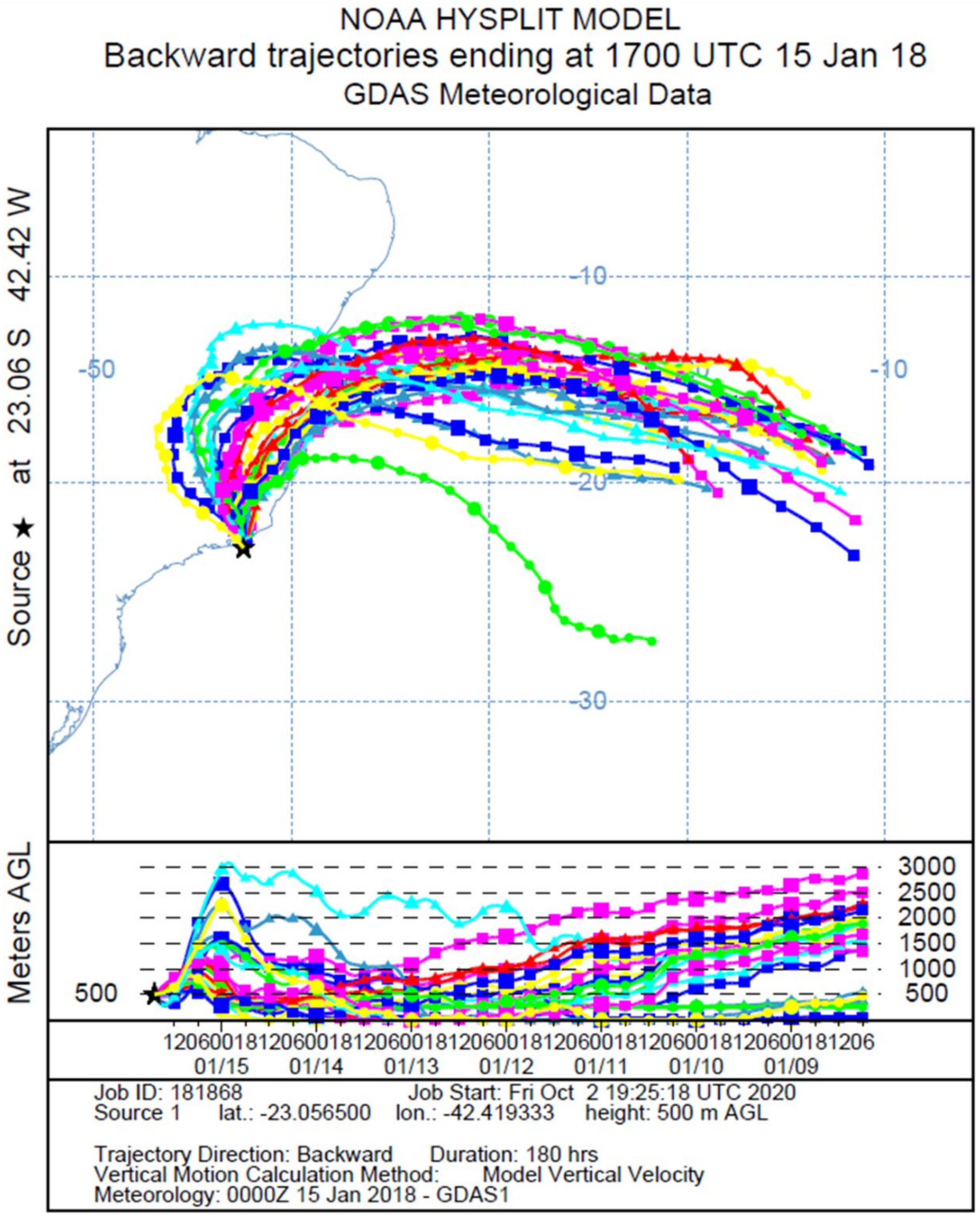

Figure S4. Backward air trajectories (72 hour) computed for sampling point #16.1 using NOAA's HYSPLIT Model.

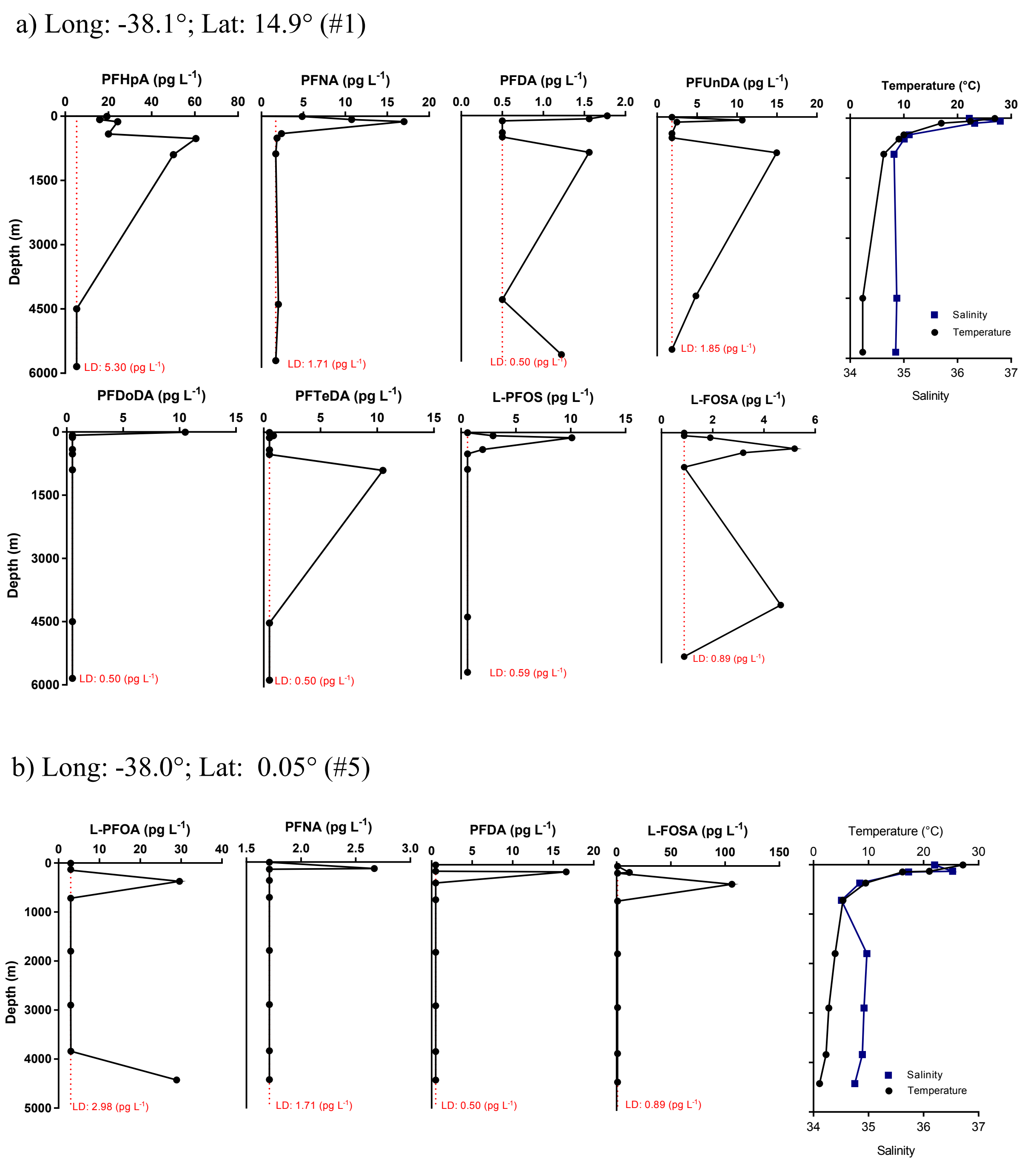

Figure S5. Vertical profiles of PFAS (pg L^{-1}) detected above MDL in ocean water columns from Tropical Atlantic Ocean (#1 (a) and #5 (b)) together with Salinity and Temperature (°C).

a) Long: -30.3°; Lat: -8° (#7)

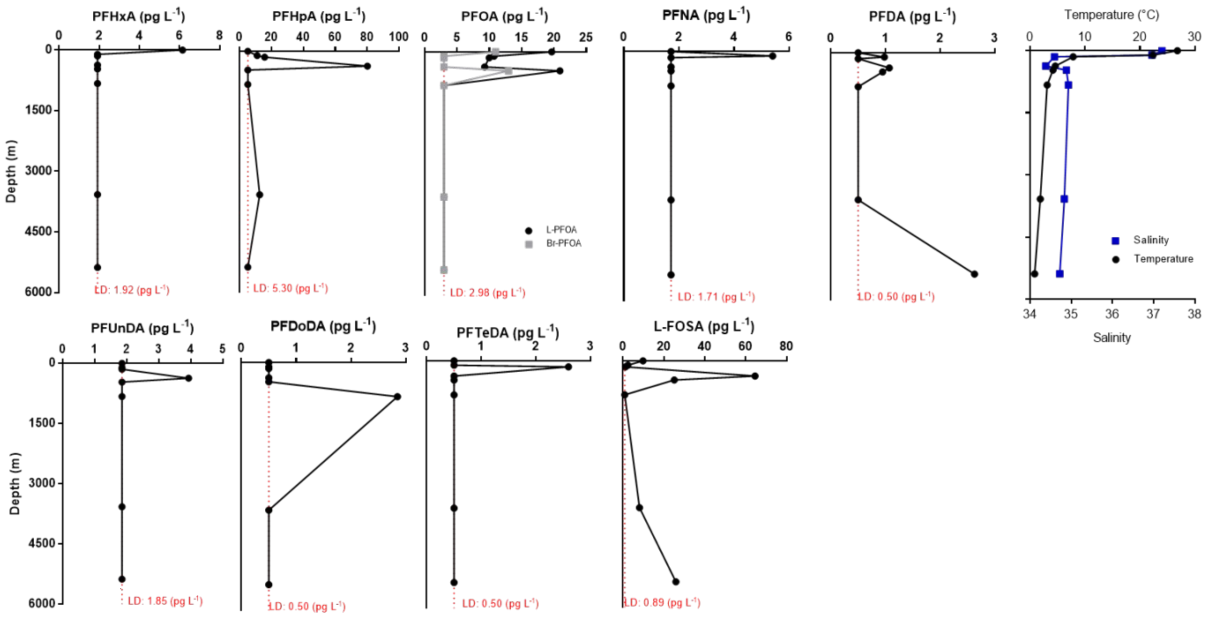

b) Long: -34.4°; Lat: -18.5° (#9)

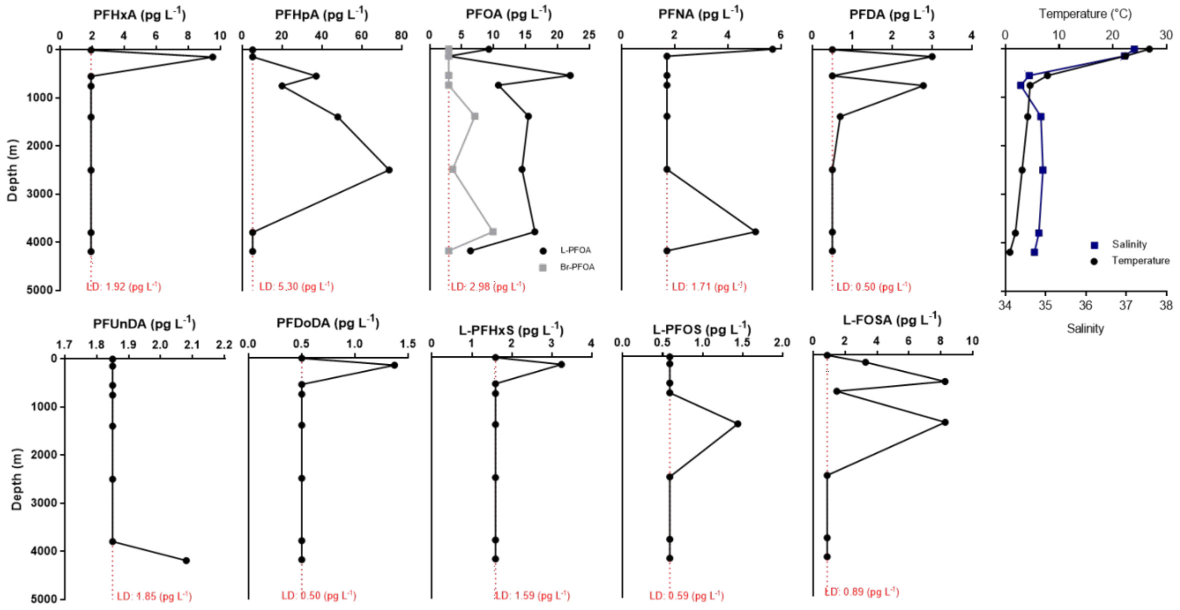

Figure S6. Vertical profiles of PFAS (pg L^{-1}) detected above MDL in ocean water columns from Tropical Atlantic Ocean (#7 (a) and #9 (b)) together with Salinity and Temperature (°C).

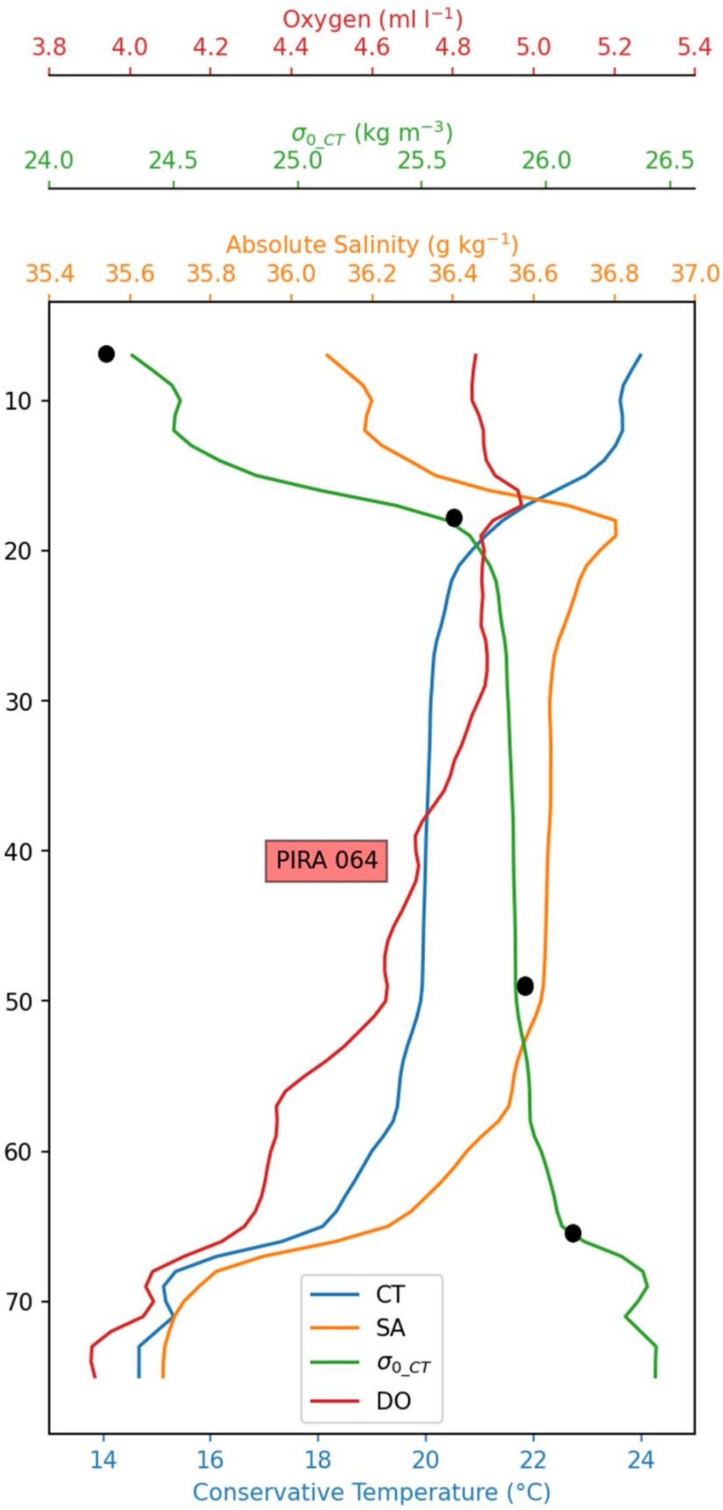

Figure S7. Vertical profile for conservative temperature, absolute salinity, oxygen, and dissolved oxygen for the sampled profile at 23ºS (#15). Black dots represent the depths where seawater samples were collected.

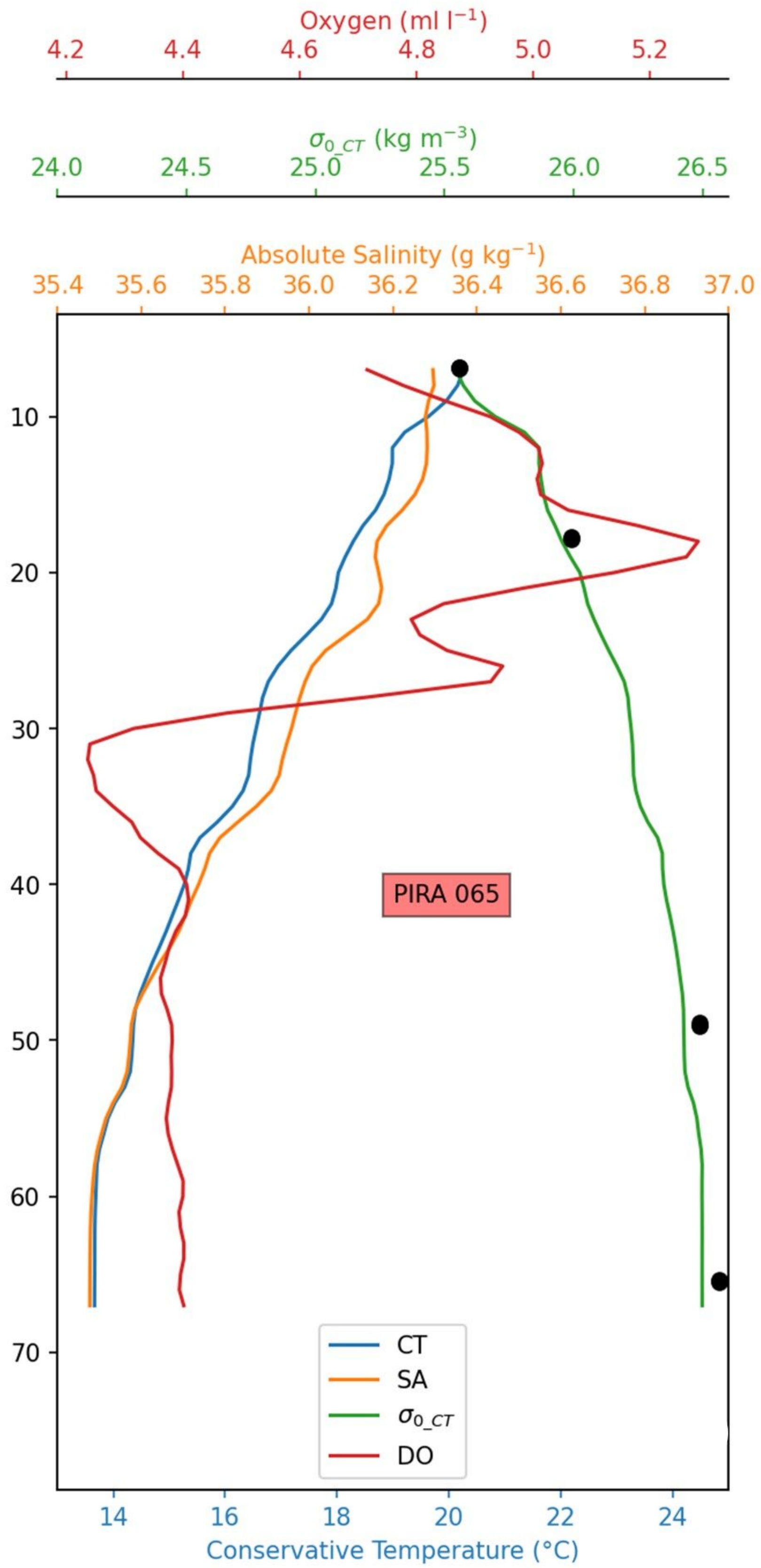

Figure S8. Vertical profile for conservative temperature, absolute salinity, oxygen, and dissolved oxygen for the sampled profile at 23ºS (#16). Black dots represent the depths where seawater samples were collected.

a) Long: -41.6°; Lat: -23.0° (#15)

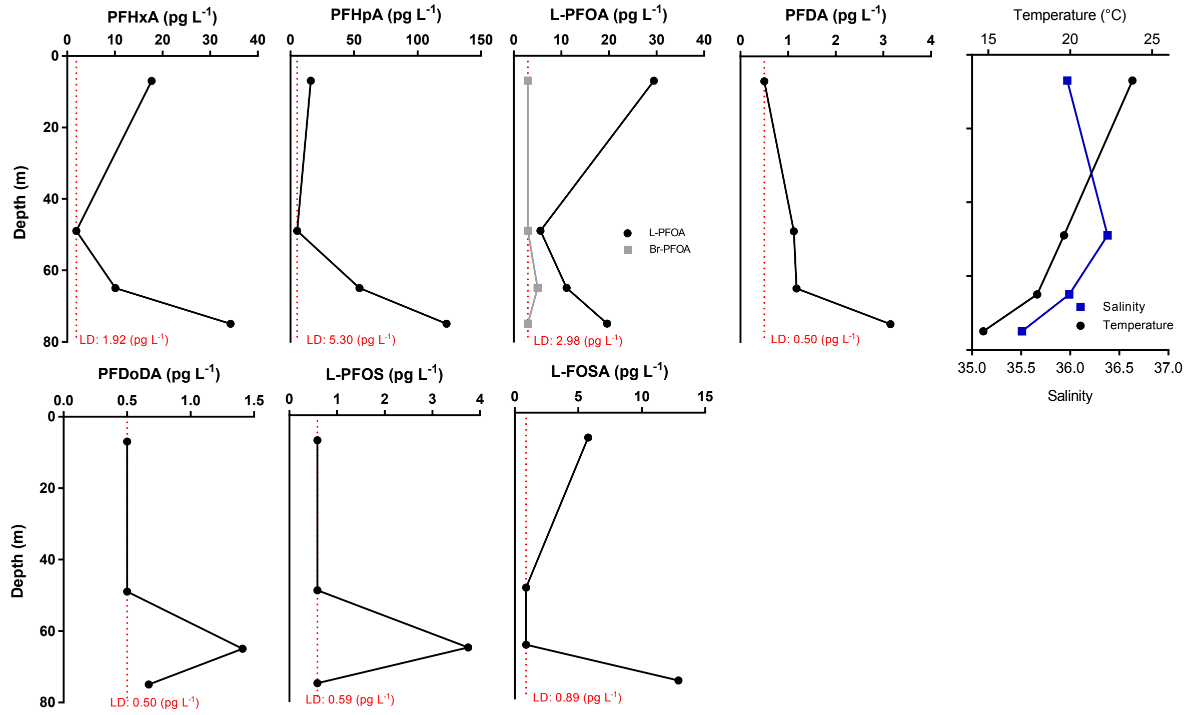

b) Long: -42.4°; Lat: -23.1° (#16)

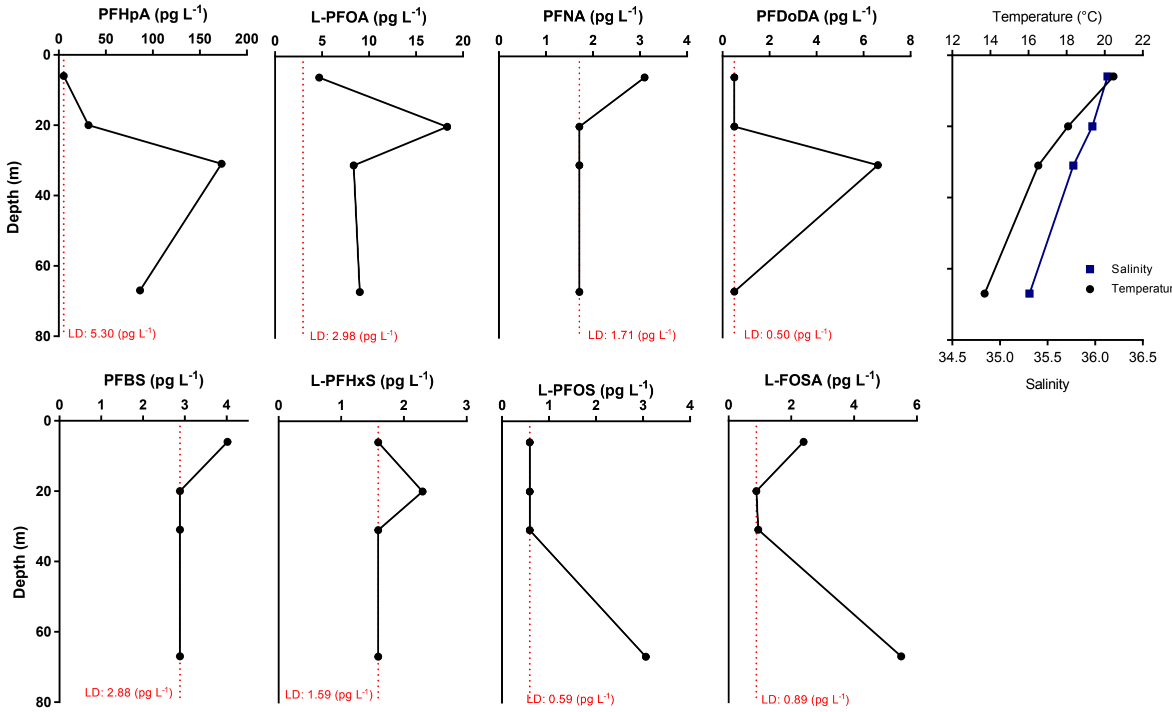

Figure S9. Vertical profiles of PFAS (pg L^{-1}) detected above MDL in ocean water columns from Rio de Janeiro upwelling (#15 (a) and #16 (b)) together with Salinity and Temperature (°C).

Reference

1. Nascimento, R. A. *et al.* Sulfluramid use in Brazilian agriculture: A source of per- and polyfluoroalkyl substances (PFASs) to the environment. *Environ. Pollut.* **242**, 1436–1443 (2018).
2. Stramma, L. & England, M. On the water masses and mean circulation of the South Atlantic Ocean. *J. Geophys. Res.* **104**, 863–883 (1999).
3. Liu, M. & Tanhua, T. Characteristics of Water Masses in the Atlantic Ocean based on GLODAPv2 data. *Ocean Sci. Discuss.* **2019**, 1–43 (2019).
4. Emílsson, I. The shelf and coastal waters off southern Brazil . *Boletim do Instituto Oceanográfico* vol. 11 101–112 (1961).
5. Miranda, L. B. de. Forma da correlação T-S de massas de água das regiões costeira e oceânica entre o Cabo de São Tomé (RJ) e a Ilha de São Sebastião (SP), Brasil . *Boletim do Instituto Oceanográfico* vol. 33 105–119 (1985).
6. Sverdup, H, U., Fleming, M. W., H., J. & Richard. The Oceans: Their Physics, Chemistry, and General Biology. *Q. J. R. Meteorol. Soc.* **70**, 159–160 (1944).
7. Silveira, I. C. A. da, Schmidt, A. C. K., Campos, E. J. D., Godoi, S. S. de & Ikeda, Y. A corrente do Brasil ao largo da costa leste brasileira . *Revista Brasileira de Oceanografia* vol. 48 171–183 (2000).
8. Bashmachnikov, I., Nascimento, Â., Neves, F., Menezes, T. & Koldunov, N. V. Distribution of intermediate water masses in the subtropical northeast Atlantic. *Ocean Sci.* **11**, 803–827 (2015).
9. Ferreira, M. L. de C. & Kerr, R. Source water distribution and quantification of North Atlantic Deep Water and Antarctic Bottom Water in the Atlantic Ocean. *Prog. Oceanogr.* **153**, 66–83 (2017).
10. Talley, L. D. Closure of the global overturning circulation through the Indian, Pacific, and Southern Oceans: Schematics and transports. *Oceanography* **26**, 80–97 (2013).
11. González-Gaya, B., Dachs, J., Roscales, J. L., Caballero, G. & Jiménez, B. Perfluoroalkylated Substances in the Global Tropical and Subtropical Surface Oceans. *Environ. Sci. Technol.* **48**, 13076–13084 (2014).
12. Benskin, J. P., Li, B., Ikonomou, M. G., Grace, J. R. & Li, L. Y. Per- and Poly fl uoroalkyl Substances in Land fi ll Leachate: Patterns, Time Trends, and Sources. *Environ. Sci. Technol.* **46**, 11532–11540 (2012).
13. Yamashita, N. *et al.* Perfluorinated acids as novel chemical tracers of global circulation of ocean waters. *Chemosphere* **70**, 1247–1255 (2008).
14. Zhao, Z. *et al.* Distribution and long-range transport of polyfluoroalkyl substances in the Arctic, Atlantic Ocean and Antarctic coast. *Environ. Pollut.* **170**, 71–77 (2012).
15. Yeung, L. W. Y. *et al.* Vertical Profiles, Sources, and Transport of PFASs in the Arctic Ocean. *Environ. Sci. Technol.* **51**, 6735–6744 (2017).
16. Cai, M. *et al.* Per- and polyfluoroalkyl substances in snow, lake, surface runoff water and coastal seawater in Fildes Peninsula, King George Island, Antarctica. *J. Hazard. Mater.*

209–210, 335–342 (2012).

17. Wei, S. *et al.* Distribution of perfluorinated compounds in surface seawaters between Asia and Antarctica. *Mar. Pollut. Bull.* **54**, 1813–1818 (2007).

18. Ahrens, L., Xie, Z. & Ebinghaus, R. Distribution of perfluoroalkyl compounds in seawater from Northern Europe, Atlantic Ocean, and Southern Ocean. *Chemosphere* **78**, 1011–1016 (2010).

19. Ahrens, L., Barber, J. L., Xie, Z. & Ebinghaus, R. Longitudinal and Latitudinal Distribution of Perfluoroalkyl Compounds in the Surface Water of the Atlantic Ocean. *Environ. Sci. Technol.* **43**, 3122–3127 (2009).

20. Benskin, J. P. *et al.* Perfluoroalkyl Acids in the Atlantic and Canadian Arctic Oceans. *Environ. Sci. Technol.* **46**, 5815–5823 (2012).
